# Protein Kinase C Regulates Human Pluripotent Stem Cell Self-Renewal

**DOI:** 10.1371/journal.pone.0054122

**Published:** 2013-01-21

**Authors:** Masaki Kinehara, Suguru Kawamura, Daiki Tateyama, Mika Suga, Hiroko Matsumura, Sumiyo Mimura, Noriko Hirayama, Mitsuhi Hirata, Kozue Uchio-Yamada, Arihiro Kohara, Kana Yanagihara, Miho K. Furue

**Affiliations:** 1 Laboratory of Stem Cell Cultures, Department of Disease Bioresources Research, National Institute of Biomedical Innovation, Ibaraki, Osaka, Japan; 2 Laboratory of Cell Cultures, Department of Disease Bioresources Research, National Institute of Biomedical Innovation, Ibaraki, Osaka, Japan; 3 Laboratory of Animal Models for Human Diseases, Department of Disease Bioresources Research, National Institute of Biomedical Innovation, Ibaraki, Osaka, Japan; Kanazawa University, Japan

## Abstract

**Background:**

The self-renewal of human pluripotent stem (hPS) cells including embryonic stem and induced pluripotent stem cells have been reported to be supported by various signal pathways. Among them, fibroblast growth factor-2 (FGF-2) appears indispensable to maintain self-renewal of hPS cells. However, downstream signaling of FGF-2 has not yet been clearly understood in hPS cells.

**Methodology/Principal Findings:**

In this study, we screened a kinase inhibitor library using a high-throughput alkaline phosphatase (ALP) activity-based assay in a minimal growth factor-defined medium to understand FGF-2-related molecular mechanisms regulating self-renewal of hPS cells. We found that in the presence of FGF-2, an inhibitor of protein kinase C (PKC), GF109203X (GFX), increased ALP activity. GFX inhibited FGF-2-induced phosphorylation of glycogen synthase kinase-3β (GSK-3β), suggesting that FGF-2 induced PKC and then PKC inhibited the activity of GSK-3β. Addition of activin A increased phosphorylation of GSK-3β and extracellular signal-regulated kinase-1/2 (ERK-1/2) synergistically with FGF-2 whereas activin A alone did not. GFX negated differentiation of hPS cells induced by the PKC activator, phorbol 12-myristate 13-acetate whereas Gö6976, a selective inhibitor of PKCα, β, and γ isoforms could not counteract the effect of PMA. Intriguingly, functional gene analysis by RNA interference revealed that the phosphorylation of GSK-3β was reduced by siRNA of PKCδ, PKCε, and ζ, the phosphorylation of ERK-1/2 was reduced by siRNA of PKCε and ζ, and the phosphorylation of AKT was reduced by PKCε in hPS cells.

**Conclusions/Significance:**

Our study suggested complicated cross-talk in hPS cells that FGF-2 induced the phosphorylation of phosphatidylinositol-3 kinase (PI3K)/AKT, mitogen-activated protein kinase/ERK-1/2 kinase (MEK), PKC/ERK-1/2 kinase, and PKC/GSK-3β. Addition of GFX with a MEK inhibitor, U0126, in the presence of FGF-2 and activin A provided a long-term stable undifferentiated state of hPS cells even though hPS cells were dissociated into single cells for passage. This study untangles the cross-talk between molecular mechanisms regulating self-renewal and differentiation of hPS cells.

## Introduction

The self-renewal of human pluripotent stem (hPS) cells including embryonic stem (hES) and induced pluripotent stem (hiPS) cells have been reported to be supported by various signal pathways, including transforming growth factor-β/activin A/Nodal [1]–[3], sphingosine-1-phosphate/platelet derived growth factor (S1P/PDGF) [4], insulin growth factor (IGF)/insulin [5] and fibroblast growth factor-2 (FGF-2) [6]–[9]. The process of self-renewal appears to be regulated synergistically through the various pathways via growth factor or cytokine supplementation. Among them, FGF-2 signaling appears indispensable to hPS cells [10]–[12].

FGF family members including FGF-2, bind to FGF receptors (FGFRs) and induce activation of the mitogen-activated protein kinase/extracellular signal-regulated kinase-1/2 (ERK-1/2) kinase (MEK), phosphatidylinositol-3 kinase (PI3K), and phospholipase C-γ (PLC-γ)/protein kinase C (PKC) pathways [13]. MEK-1/2 activation by FGFR results in ERK-1/2 phosphorylation, which subsequently translocates into the nucleus leading to phosphorylation of transcription factors such as c-Myc, c-Jun, and c-Fos. PI3K, a lipid kinase activates pleckstrin homology (PH) domain containing proteins such as AKT, and 3-phosphoinositide-dependent kinase-1 (PDK1). AKT directly activates murine double minute 2 (MDM2), a negative regulator of p53. p53 is responsible for DNA damage surveillance and in response initiates cell cycle arrest and DNA repair. Interestingly, AKT also inhibits glycogen synthase kinase-3 (GSK-3), a negative regulator of Wnt signaling by phosphorylation [14]. However, the contributions of FGF-2 downstream pathways in the self-renewal of hPS cells have been controversial [9], [14]–[18]. The ERK pathway has been thought to promote cell proliferation and adhesion but also differentiation in hES cells. The PI3K pathway plays important roles in proliferation, differentiation, survival, and cellular transformation.

Previously, we found that a proteoglycan, heparin promotes FGF-2 activity on the growth of undifferentiated hES cells in a minimal growth factor-defined culture medium, hESF9 [8], in which the effect of exogenous factors can be analyzed without the confounding influences of undefined components [8], [19]–[23] because insulin, transferrin, albumin conjugated with oleic acid, and FGF-2 (10 ng/ml) are the only protein components. Understanding cell signaling in undifferentiated hPS cells has lead to the development of optimal conditions for culturing hPS cells. However, manipulation of hPS cells still remains difficult because hPS cells as a single cell are unstable of self-renewal. Although Rho-associated kinase (ROCK) inhibitor (Y-27632) is quite effective to markedly diminish dissociation-induced apoptosis of single cells of hPS cells [24], the continuous use of the ROCK inhibitor increases differentiated cells [25]. For developing application using hPS cells, such as cell based therapy or toxicity screening tests, handling cell numbers would be beneficial. Even for basic research, handling cell numbers would be useful when the cells are dissociated for passages or differentiation. Presumably, if the culture conditions were able to fully support undifferentiated state, even single cells might maintain undifferentiated state. We suspected that there were unrevealed mechanisms to maintain undifferentiated state of single hPS cells. To further understand FGF-2 related molecular mechanisms regulating self-renewal would enhance understanding unclarified cell signaling in hPS cells. Therefore, we screened a kinase inhibitor library using a high-throughput alkaline phosphatase (ALP) activity-based assay in a minimal growth factor-defined culture medium, hESF9. We found that in the presence of FGF-2, an inhibitor of PKCs, GF109203X (GFX), increased ALP activity, suggesting that PKC reduces self-renewal of hPS cells. GFX inhibited FGF-2-induced GSK-3β phosphorylation. Addition of activin A increased phosphorylation of GSK-3β and ERK-1/2 synergistically with FGF-2 whereas activin A alone did not induce phosphorylation of GSK-3β. GFX negated differentiation of hPS cells induced by a PKC activator, phorbol 12-myristate 13-acetate (PMA) whereas Gö6976, a selective inhibitor of PKCα, β, and γ isoforms did not counteract the effect of PMA. Functional gene analysis by RNA interference revealed that siRNA of PKCδ, ε, and ζ isoforms decreased phosphorylation of GSK-3β and also siRNA of PKCε and ζ isoforms decreased phosphorylation of ERK-1/2 in hPS cells. siRNA of PKCε decreased phosphorylation of AKT. On the basis of these results, we suggest that PKCδ, ε and ζ isoforms are FGF-2 downstream effectors, and they play various roles in regulating hPS cell self-renewal. This study helps to untangle the cross-talk between molecular mechanisms regulating self-renewal and differentiation of hPS cells.

## Results

### PKC inhibitor increased ALP activity of hiPS cells

**Figure 1:**
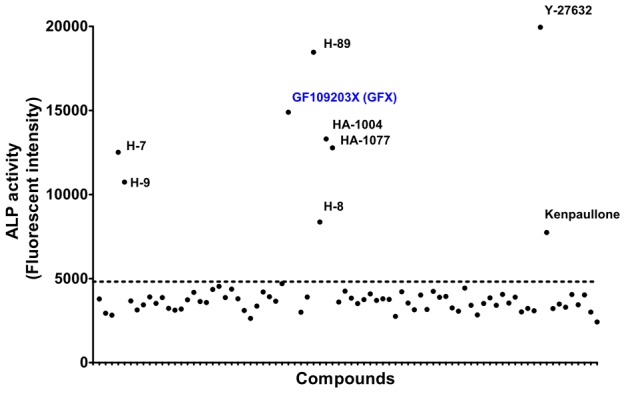
An ALP activity-based high-throughput screening assay of chemical library for PKC inhibitors. The ALP activity using 4-methylumbelliferyl phosphate [59] in 201B7 hiPS cells in a 96-well plate was measured by fluorometry. Each dot on the graph represents the fluorescent intensity for each compound of the kinase inhibitor library. Dotted line indicates the level for DMSO as a control.

Previously, we detected the cell proliferative effect of heparin on hES cells without feeder cells in a minimal growth factor-defined culture medium, hESF9 [8], in which the effect of exogenous factors can be analyzed without the confounding influences of undefined components [8], [19]–[23]. In this culture condition using hESF9 medium (Table S1) on bovine fibronectin (FN), a high-throughput ALP activity-based assay was performed to evaluate a library of chemical kinase inhibitors to understand FGF-2 related molecular mechanisms regulating self-renewal of hPS cells. Nine compounds were found to increase ALP activity of the hiPS cell line 201B7 [26] (Fig. 1): Kenpaullone, which is a substitute for a reprogramming factor KLF-4 in mouse iPS cells [27]; Y-27632, which is a Rho-kinase (ROCK) inhibitor known to enhance hES cells survival [24]; HA-1004, H-89, and HA-1077, which are kinase inhibitors presumed to target ROCK [28]; GF109203X (GFX) [29], which is a inhibitor for PKC isoforms; and H-7, H-8, and H-9, which are also thought to target PKC [30]. These results suggest that FGF-2 induces PKC, and PKC acts downstream of FGF-2 to regulate self-renewal of hPS cells.

### Effect of PKC inhibitor on FGF-2 signaling in hPS cells

**Figure 2:**
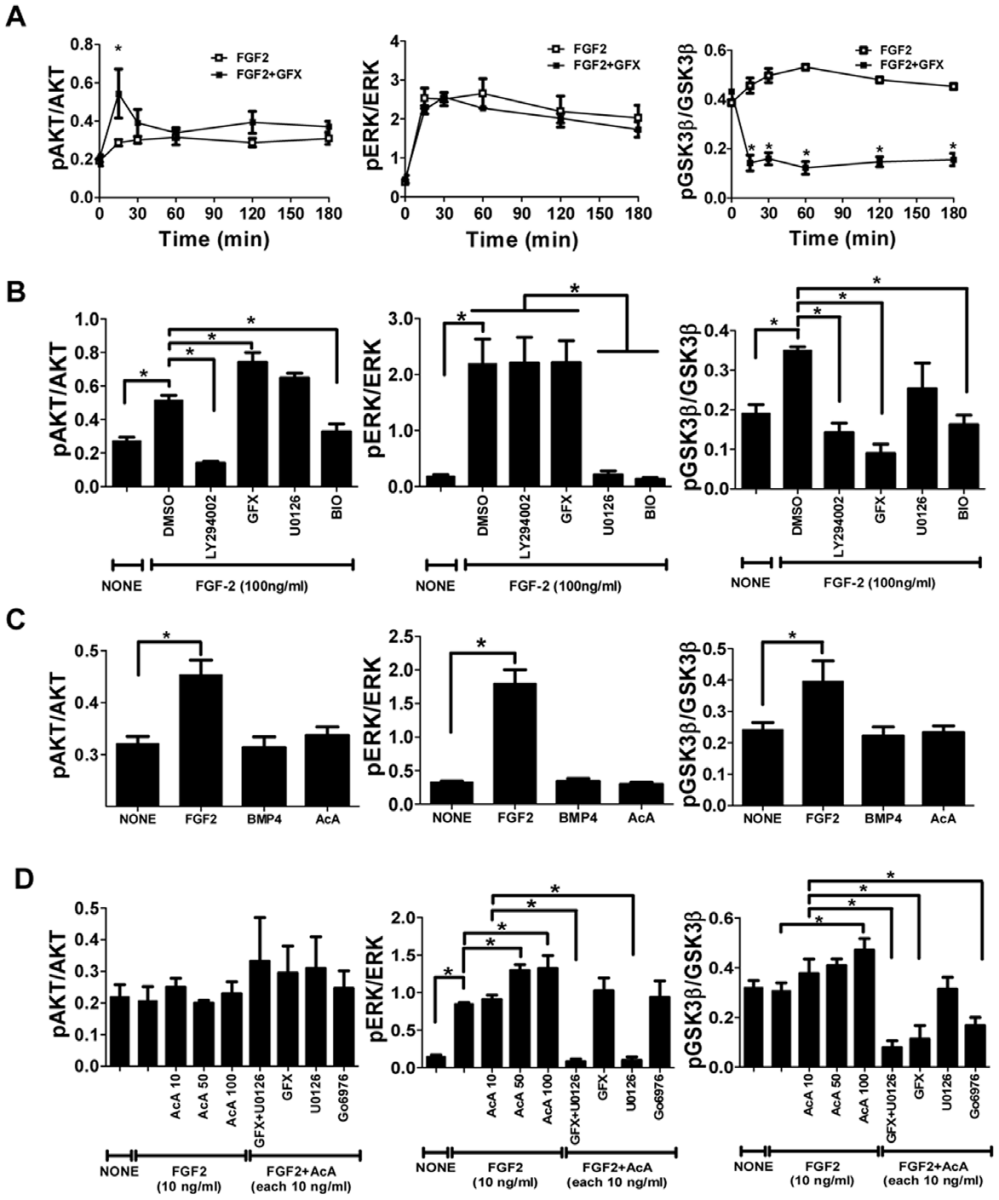
Effect of PKC inhibitor on FGF-2 signaling in hPS cells. The phosphorylation levels in H9 hES cells were measured by AlphaScreen® SureFire® assay kit. The values of the y-axis are the ratio of each phosphorylation to each total signal protein. (A) The cells were stimulated with FGF-2 (100 ng/ml) in fresh medium without insulin after overnight starvation and incubated with (open square) or without GFX (5 µM, closed square) for 180 minutes. The data are represented as means ± SE (n = 3). *P<0.05. (B) The cells were stimulated with FGF-2 (100 ng/ml) in fresh medium without insulin after overnight starvation. Fifteen minutes after FGF-2 addition together with each inhibitor as indicated on the panel. The data are represented as means ± SE (n = 3). *P<0.05. (C) The cells were treated with FGF-2 (100 ng/ml), BMP-4 (100 ng/ml) or activin A (100 ng/ml) in fresh medium without insulin after overnight starvation. Fifteen minutes after the addition of each growth factor as indicated on the panel. The data are represented as means ± SE (n = 3). *P<0.05. (D) The cells after growth factor starvation were stimulated with FGF-2 (10 ng/ml) and activin A (10 or 100 ng/ml) together with U0126 (5 µM) and GFX (5 µM) or Gö6976 (5 µM) in fresh medium without insulin for 15 minutes. Fifteen minutes after the addition of each growth factor/inhibitor as indicated on the panel. The data are represented as means ± SE (n = 3). *P<0.05.

To examine how GFX influenced FGF-2 signaling in hPS cells, the phosphorylation of AKT, ERK-1/2, and GSK-3β induced by FGF-2 with GFX was confirmed by western blotting analysis (Fig. S1A, S1B, S1C, S1D). Then, the phosphorylation levels were quantified by AlphaScreen® SureFire® assay kit. Human ES cells H9 [31] after starvation of FGF-2 and insulin were treated with FGF-2 with and without GFX. FGF-2 significantly stimulated the phosphorylation of AKT, ERK-1/2, and GSK-3β in H9 cells in 15 minutes (Fig. 2A, 2B, 2C) as described previously [16], [32]. Addition of GFX at 5.0 µM in the presence of FGF-2 significantly increased AKT phosphorylation in 15 minutes compared with addition of FGF-2 alone (Fig. 2A, 2B, Fig. S1E). The level of ERK-1/2 phosphorylation induced by FGF-2 with GFX was comparable with that without GFX (Fig. 2A). On the other hand, FGF-2-induced GSK-3β phosphorylation was completely inhibited by GFX (Fig. 2A, 2B) at concentrations higher than 1 µM treatment (Fig. S1E).

Addition of the PI3K inhibitor LY-294002 with FGF-2 completely inhibited AKT phosphorylation and significantly reduced GSK-3β phosphorylation (Fig. 2B, Fig. S1B). Addition of the MEK inhibitor U0126 with FGF-2 reduced ERK-1/2 phosphorylation and had little influence on GSK-3β phosphorylation. Addition of the GSK inhibitor BIO with FGF-2 significantly reduced phosphorylation of not only AKT, but also ERK-1/2 and GSK-3β.

Neither BMP-4 nor activin A in the absence of FGF-2 induced the phosphorylation of AKT, ERK-1/2, or GSK-3β in 201B7 iPS cells (Fig. 2C, Fig. S1C). From our previous report that activin A acts synergistically with FGF-2 in stimulating the phosphorylation of ERK-1/2 [20], we speculated that activin A may increase the phosphorylation of GSK-3β synergistically with FGF-2. Addition of increasing concentrations of activin A with FGF-2 increased phosphorylation of both GSK-3β and ERK-1/2 in a dose-dependent manner in H9 hES cells (Fig. 2D, Fig. S1D). Addition of U0126 with FGF-2 and activin A had little influence on phosphorylation of both AKT and GSK-3β, and completely inhibited phosphorylation of ERK-1/2. Addition of GFX together with U0126 in the presence of FGF-2 and activin A not significantly increased phosphorylation of AKT, while it completely inhibited phosphorylation of both ERK-1/2 and GSK-3β (Fig. 2D, Fig. S1D). A selective inhibitor of classical PKC (α, β, and γ isoforms) [29], Gö6976 had little influence on phosphorylation of AKT and decreased phosphorylation of GSK-3β less than GFX. These results suggested that FGF-2-induced PKC stimulated phosphorylation of GSK-3β and that GFX inhibited the PKC-induced phosphorylation of GSK-3β, but it increased phosphorylation of AKT (Fig. S2).

### Effect of GFX and PMA on colony morphology of the cells

**Figure 3:**
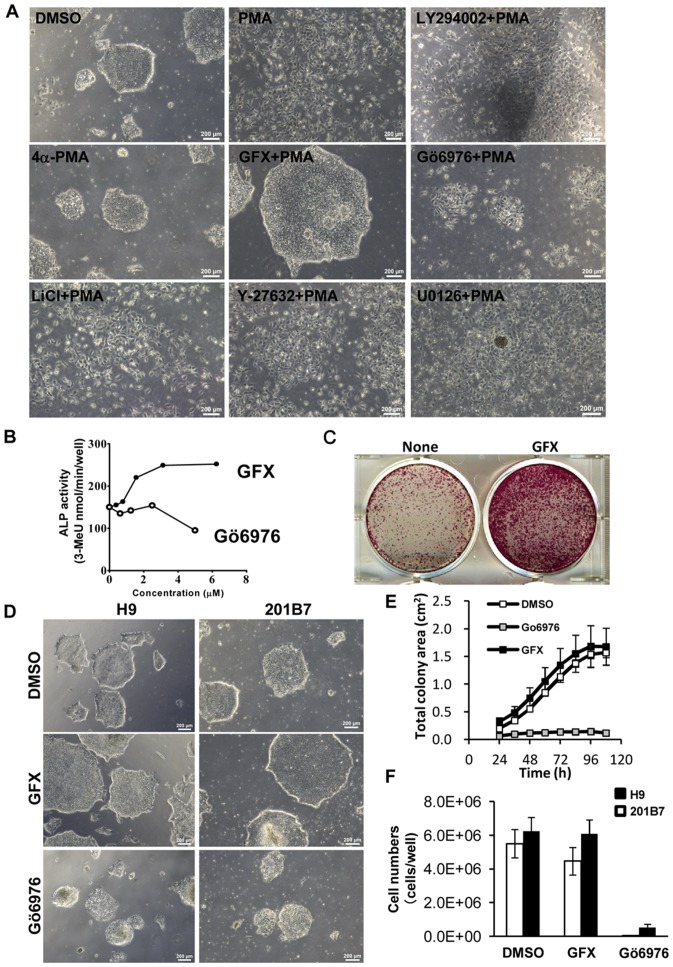
The effect of PKC on the morphologies of hPS cells with or without GFX. (A) Phase-contrast image of 201B7 hiPS cells cultured in feeder-free hESF9 defined medium on FN 24 hours after treatment with DMSO, PMA (10 nM), 4α-PMA (10 nM), GFX (5 µM), PMA (10 nM) with GFX (5 µM), PMA (10 nM) with Gö6976 (5 µM), PMA (10 nM) with LY-294002 (50 µM), PMA (10 nM) with LiCl (1 mM), PMA (10 nM) with Y-27632 (10 µM), or PMA (10 nM) with U0126 (20 µM). An inactive PMA analogue, 4α-PMA is used as negative control. Scale bars, 200 µm. (B) Quantitative ALP-based assay of 201B7 hiPS cells cultured in feeder-free hESF9 medium with GFX (closed circle) or Gö6976 (open circle) as indicated concentrations. (C) Colony forming efficiency of dissociated single hPS cells cultured with or without GFX. Dissociated single 201B7 cells seeded at 250,000 cells/well were grown on a 6-well plate coated with FN (2 µg/cm^2^) in hESF9 medium supplemented with and without 1 µM GFX. A in 5 days and stained with ALP fast-red substrate. (D) Phase-contrast image of 201B7 hiPS cells or H9 hES cells cultured in feeder-free hESF9 medium with DMSO (open square), GFX (5 µM, gray square), or Gö6976 (5 µM, closed square). (E) Growth of cell colony area of hPS cells in the presence of GFX or Gö6976. The whole images of 201B7 cell colonies grown in a 6-well-plate coated with FN in the presence of DMSO, GFX or Gö6976 in hESF9 medium was measured by an analysis software, Cell-Quant. The images were captured every 12 hours in live cell imaging system Biostation CT. The data are represented as means ± SD (n = 3). (**F**) Cell growth of hPS cells in the presence of GFX or Gö6976. The numbers of H9 (open bars) and 201B7 cells (closed bars) grown in a 6-well-plate coated with FN in the presence of DMSO, GFX or Gö6976 in hESF9 medium were counted on 5 days. The data are represented as means ± SD (n = 3).

To confirm the speculation that PKCs play roles in regulating self-renewal in hPS cells, the effect of the PKC activator PMA with several kinase inhibitors on the culture of 201B7 hiPS cells was determined (Fig. 3A). Treatment with PMA scattered the iPS cell colony dramatically. PMA-treatment with LY-294002, lithium chloride (LiCl, GSK inhibitor), Y-27632, or U0126 did not reverse the morphological change whereas GFX negated the effect of PMA on cultured 201B7 cells. Gö6976 did not negate the effect of PKC. The effect of Gö6976 was compared with that of GFX on ALP-activity of the cells: GFX with FGF-2 increased the ALP-activity of 201B7 iPS cells, while Gö6976 with FGF-2 had little effect on ALP-activity of the cells (Fig. 3B). GFX increased colony forming efficiency in hESF9 medium (Fig. 3C). Gö6976 did not increase the colony sizes of 201B7 cells and also cell numbers of H9 and 201B7 cells whereas GFX increased the colony sizes and also cell numbers (Fig. 3D, 3E, 3F). PMA activates PKCα, β, γ, δ, ε, η, and θ whereas GFX inhibits PKCα, β, γ, δ, ε, and ζ isoforms. Gö6976 inhibits PKCα, β, and γ isoforms. These results and findings suggested that PKCδ or ε isoforms regulate undifferentiated state of hPS cells.

### Isoform-specific function of PKCs in FGF-2 signaling

**Figure 4:**
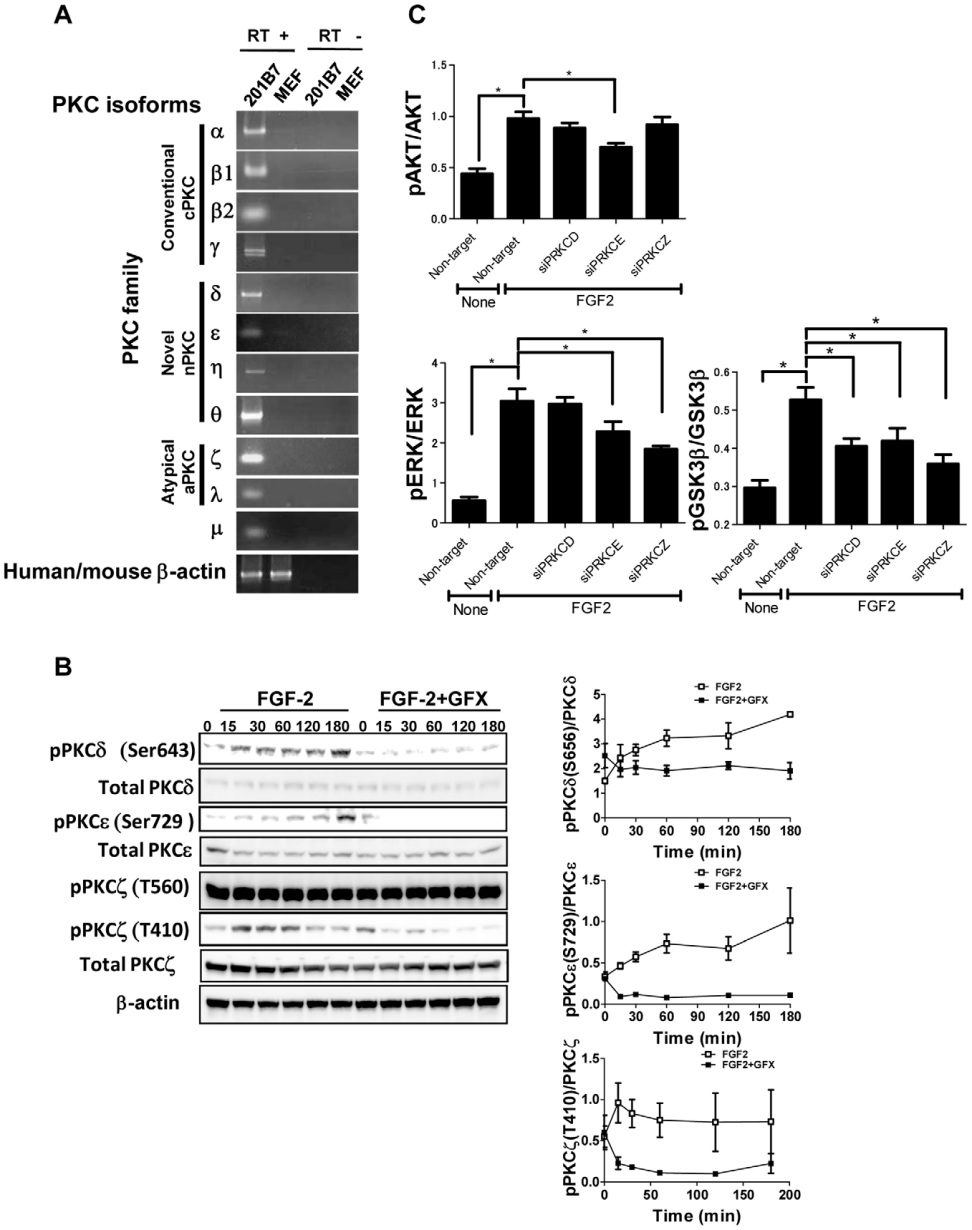
Specific-isoform of PKCs function in FGF-2 signaling. (**A**) RT-PCR analysis of PKC isoform expression. Total RNA was extracted from the undifferentiated 201B7 hiPS cells cultured on feeder cells (CF-1) with KSR-based medium or the feeder cells. Primers were listed in Table S3. (**B**) Phosphorylation of PKCδ, ε, or ζ isoforms induced by FGF-2 (open square) with GFX (closed square). 201B7 hiPS cells were stimulated with FGF-2 (100 ng/ml) after overnight starvation and incubated with or without GFX (5 µM) for 180 minutes. The cells were lysed and followed by western blot analysis using an antibody detecting the phosphorylation or total protein amount of PKCδ, PKCε, or PKCζ. Protein content quantified from the gel blot images (n = 3). The values of the y-axis are the ratio of each phosphorylation to each total signal protein. (**C**) FGF-2 signaling in hPS cells with specific PKC isoforms-targeting siRNA. 201B7 iPS cells were transfected with specific PKCδ, ε, or ζ isoforms-targeting siRNA or non-targeting siRNA. The phosphorylation levels of the cells treated with FGF-2(100 ng/ml) after overnight starvation were measured by AlphaScreen® SureFire® assay kit. The values of the y-axis are the ratio of each phosphorylation to each total signal protein. The data are represented as means ± SE (n = 3). *P<0.05.

To determine the isoform-specific function of PKCs on FGF-2 signaling, at first the expression of 11 PKC isoform genes in 201B7 iPS cells was determined by RT-PCR. The results showed that the cells expressed all of 11 PKC isoforms examined here (Fig. 4A). The PKC inhibitor results described above suggested that PKCδ or PKCε might be responsible for GSK-3β phosphorylation but there is a possibility that PKCζ might also be involved. Then, we examined whether FGF-2 stimulated phosphorylation of PKCδ, PKCε or PKCζ with or without GFX. Image analysis of western blotting showed that the phosphorylation of PKCδ and PKCε was increased in a time-dependent manner after stimulation of FGF-2 and the phosphorylation of PKCζ was increased in 15 min after stimulation of FGF-2 and then decreased, suggesting that activation mechanism of PKCζ might be related with GSK-3β phosphorylation (Fig. 4B). GFX diminished the increased phosphorylation of all three PKCs. These result indicated that FGF-2 induced PKCδ, PKCε, and PKCζ in hPS cells.

We next examined the effects of short interfering RNA (siRNA) targeting PKCδ, PKCε or PKCζ on FGF-2 signaling in 201B7 iPS cells. The efficacy and specificity of siRNA was confirmed by quantitative RT-PCR (Fig. S3A). The expression of the targeted PKC genes was inhibited for at least 60%. The phosphorylation levels of AKT, ERK-1/2 and GSK-3β were measured in these PKCs-knockdown cells by AlphaScreen® SureFire® assay kit. The results showed that knockdown of PKCδ, and PKCζ did not affect FGF-2-induced AKT phosphorylation while knockdown of PKCε significantly reduced it (Fig. 4C). Knockdown of either PKCε or PKCζ isoform significantly decreased FGF-2-induced ERK-1/2 phosphorylation. GFX which is reported to target PKCα, β, γ, δ, ε and ζ isoforms did not change the level of FGF-2-induced ERK-1/2 phosphorylation, as shown above (Fig. 2 and Fig. S1). These results implied that cross-interaction among PKC isoforms might affect on the level of FGF-2-induced ERK-1/2 phosphorylation. Then, the cells were treated with the inhibitory peptide cocktail for all isoforms (PKCα, β, γ, δ, ε and ζ), or the inhibitory peptide cocktail for PKCδ, ε, and ζ. The inhibitory peptide cocktail for all isoforms did not affect on FGF-2-induced ERK-1/2 phosphorylation. On the other hand, the inhibitory peptide cocktail for PKCδ, ε, and ζ inhibited the ERK-1/2 phosphorylation (Fig. S4). These results suggested that inhibitions of all isoforms neutralized the reducing effect on FGF-2-induced ERK-1/2 phosphorylation by the inhibition of PKCε and ζ. GSK-3β phosphorylation was significantly reduced by the knockdown of all three PKC isoforms, compared with that by non-target siRNA. These results suggest that FGF-2 induced PKCs, followed by phosphorylation of ERK-1/2 and GSK-3β in hPS cells (Fig. S3B). From these results, we showed that FGF-2 induced PKCδ, ε, and ζ, resulting in stimulation of differentiation in hPS cells which might cause instability of the self-renewal state of hPS cells and that GFX targets these PKC isoforms in hPS cells, resulting in enhanced self-renewal of hPS cells.

### Stability of self-renewal of hPS cells in the presence of inhibitors of ERK-1/2 and PKC

**Figure 5:**
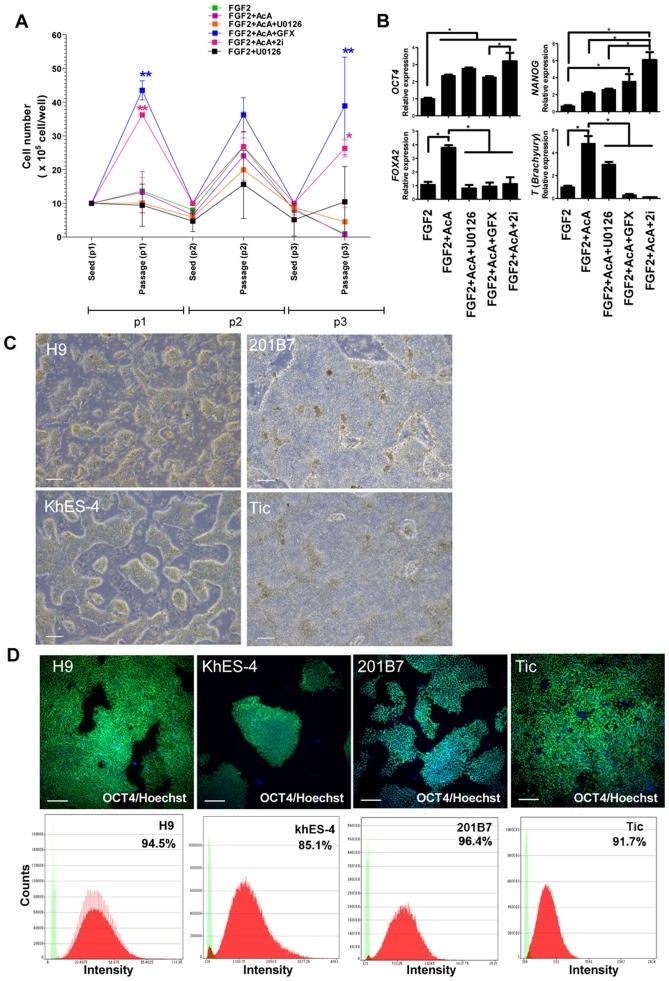
Single cell culture of hPS cells in the hESF9a_2i_ medium. (A) Cell growth of dissociated single H9 hES cells cultured in each indicated condition for three passages. Cells were reseeded at the cell density of 1×10^6^ cells/well every 5 days. When the cells were passages, cell numbers were counted. Cell growth in the hESF9a_2i_ medium was significantly different (P<0.05) from hESF9 (FGF-2), FGF-2 + activin A, FGF-2 + activin A + U0126. Cell growth in hESF9a + GFX was significantly different (P<0.05) from hESF9 (FGF-2), FGF-2 + activin A, FGF-2 + activin A + U0126, and FGF2 + U0126. The data are represented as means ± SE (n = 3). (**B**) Gene expression in the hPS cells cultured in each indicated condition for three passages. The gene expression levels of NANOG, OCT3/4, FOXA2, T in the cells were measured by real-time RT-PCR. On the y axis, the gene expression level in the cells cultured with FGF-2 in a experiment was taken as 1.0. The data are represented as means ± SE (n = 3). *P<0.05. (**C**) Phase-contrast image of hPS cells grown on FN in hESF9a_2i_ medium for 3 passages. The cells were dissociated into single cells for passage, and reseeded at a ratio of 1∶3 - 1∶5 every five days. Scale bars, 200 µm. (**D**) OCT3/4 expression in hPS cells grown on FN in hESF9a_2i_. The cells grown in hESF9a_2i_ as described above in Figure 5C were reseeded on a 6-well-plate and cultured for 5 days. The cells stained with anti-OCT3/4 antibody were visualized with Alexa Fluor 488 (upper panels). Nuclei were stained with Hoechst 33342 (blue). Scale bars, 200 µm. Whole cell images in whole plate were captured and OCT3/4 expression profiles were analyzed by Image Analyzer (lower panels). Antigen histogram (red); control histogram (green); Y axis is cell numbers and X axis is fluorescence intensity for anti-OCT3/4 antibody.

Based on the results above, we hypothesized that inhibition of both PKC and ERK-1/2 might provide stable culture of hPS cells in our minimal defined medium hESF9 with activin A. Dissociated single hPS cells were inoculated on FN in hESF9 medium supplemented with activin A (10 ng/ml) [8], [20], U0126 (5 µM) or GFX (5 µM). When dissociated single cells were cultured in hESF9, hESF9 + activin A, hESF9 + U0126, or hESF9 + activin A + U0126, many cells died or differentiated (Fig. 5A). On the other hand, when dissociated single cells were cultured in hESF9 + activin A + GFX, or hESF9 + activin A + GFX + U0126 (2i), cells could proliferate enough to be passaged. However, usually after 3 passages, epithelial-like cells appeared in the culture of hESF9 + activin A + GFX condition (Fig. S5A). Immunocytochemical analysis by image analyzer showed that ratio of OCT3/4-positive cell population in the culture of hESF9 + activin A + GFX + U0126 (2i) condition was slightly higher than that in the culture of hESF9 + activin A + GFX (Fig. S5B and S5C). Gene expression in the cells cultured in these culture conditions was analyzed by real-time PCR (Fig. 5B). The expression of an endoderm marker, FOXA2, and a mesoderm marker, T were increased by activin A but it was significantly reduced by the addition of U0126. When the cells were cultured in hESF9 + activin A + U0126 + GFX, both FOXA2 and T were inhibited at lower level and also the undifferentiated makers, NANOG and OCT3/4 were maintained at higher ratio in the cells than those in other culture conditions. Next, the serial culture of dissociated single cells of hES H9, hES KhES4, hiPS 201B7 and hiPS Tic [33] cell lines were tested in hESF9 medium supplemented with activin A (10 ng/ml), U0126 (5 µM) and GFX (5 µM) (designated hESF9a_2i_ medium; Table S1). Dissociated single hPS cells were grown on FN in hESF9a_2i_ medium for 3 passages. Phase-contrast image showed that cell morphology seemed undifferentiated although they did not form hPS typical cell colony. OCT3/4 expression profiles were confirmed by immunofluorescence analysis using image analyzer, suggesting that the hPS cells maintained undifferentiated state. Another undifferentiated maker, TRA-1-60 expression was also confirmed in hPS cells grown in hESF9a_2i_ medium for 3 passages (Fig. S6).

Serial culture more than 10 passages of undifferentiated H9 hES cells and 201B7 hiPS were tested on FN in hESF9a_2i_ medium. Undifferentiated morphologies of 201B7 hiPS (Fig. S7A) and H9 hES colonies (Fig. S8A) were maintained for more than 30 passages using the conventional passage procedure. The growth rates of H9 hES and 201B7 hiPS cells in hESF9a_2i_ medium were similar to those of cells grown in the conventional KSR-based medium on feeders (Figs. S7B and S8B). The cells retained expression of stage-specific embryonic antigen (SSEA)-4 [34], cell surface antigens TRA-1-60 [35], TRA-1-81 [35], CD90 (Thy-1) [36], and TRA-2-54 [36] (alkaline phosphatase), but did not express SSEA-1 [37] or a neural marker A2B5 [36] (Fig. S7C, S7D and S8C, S8D). The cells retained normal karyotypes (Fig. S9A), pluripotency in vitro (Fig. S9B) and in vivo (Fig. S9C). These results confirmed that inhibition of both ERK-1/2 and PKC supported the self-renewal of hPS cells.

## Discussion

**Figure 6:**
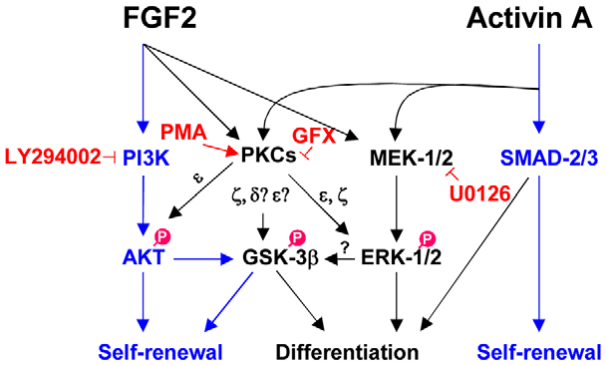
Model for the molecular mechanism of PKCs regulating self-renewal or differentiation in hPS cells. Our study suggested a model that FGF-2 activates PI3K/AKT, MEK/ERK-1/2, and PKCε/δ/ζ. PKCε, δ, and ζ inactivates directly or indirectly GSK-3β by phosphorylation which promotes differentiation of hPS cells. PKCε and ζ activates ERK-1/2 which promotes differentiation of hPS cells. Activin A activates SMAD-2/3 which controls self-renewal and differentiation while activin A together with FGF-2 activates both ERK-1/2 and PKCs. Inhibition of both ERK-1/2 and PKCs pathway provides a metastable undifferentiated state of hPS cells. Blue arrow indicated pathway promoting hPS cell self-renewal and black arrow indicated pathway promoting hPS cell differentiation.

Many studies reported that FGF-2 activates both the MAPK/ERK, and PI3K/AKT pathways, which are important for maintaining pluripotency and viability in hPS cells [9], [14]–[16]. However, FGF-2 downstream signaling is not clearly understood in hPS cells. In this study using a minimum essential defined culture system [8], [20], we showed that FGF-2 activated PI3K/AKT and MEK/ERK-1/2, but also PKCδ, ε and ζ isoforms in hPS cells (Fig. 6).

The PKC family has been implicated as an intracellular mediator of several neurotransmitters, hormones, tumor promoters, α1-adrenergic agonists, and phorbol esters, and it is important in the regulation of growth, differentiation, cell death, and neurotransmission [38]. The PKC family comprises classical (PKCα, β, and γ; activated by Ca^2+^ and phorbol esters), novel PKC (PKCδ, ε, η, and θ; activated by phorbol esters but not regulated by Ca^2+^), and atypical PKC (PKCζ and PKCι/λ; not activated by Ca^2+^ or phorbol esters). Different isoforms may perform distinct functions, as suggested by their differential pattern of localization, differences in condition of activation, and some differences in substrate specificity [39]–[40]. PKC has previously been implicated in GSK-3 regulation [41]–[42]. Fang et al. [43] showed that PKCα, βII, γ, η, and δ were capable of phosphorylating GSK-3β while PKCε and PKCζ did not phosphorylate GSK-3 by in vitro kinase assays; also, expression of constitutively active PKCα, βI, γ, η enhanced phosphorylation of cotransfected GSK-3β in HEK293 cells. On the other hand, Eng et al. [15] reported that negative construct of PKCε isoform prevented phosphorylation of GSK-3 in migrating fibroblasts._These pieces of evidence suggested that specific isoforms of PKC have different roles in different types of cells. Shuibing et al. [44] reported that activation of PKCα and/or β directs the pancreatic specification of hES cells. Recently, Feng et al. [45] reported that activation of PKCδ induces extraembryonic endoderm differentiation of hES cells. These studies suggested that PKCs might be involved in differentiation of hPS cells. Our study showed that FGF-2 induced PKCδ, ε, and ζ, resulting in phosphorylation of GSK-3β, ERK-1/2, or AKT. Chou, et al. [46] reported that the phosphorylation of PKCζ was regulated by PI3-kinase and PDK-1 in NIH 3T3 fibroblasts. Intriguingly, PKCζ can stimulate GSK-3 activity, by relieving PKB-imposed inhibition [47]. In mouse ES cells, it has been shown that PKCζ plays an important role in inducing lineage commitment in mESCs through a PKCζ–nuclear factor kappa-light-chain-enhancer of activated B cells signaling axis [48]. However, PKC inhibition does not change phosphorylation of ERK-1/2 or GSK-3β. In view of the fact that LIF mainly regulates self-renewal in mouse ES cells, isoform specific function might be cross-regulated by other signaling in the cells. Further, our study showed that the combination effect by inhibition of PKCα, β, γ, δ, ε, and ζ was different from that by inhibition of PKCε and ζ, suggesting that each PKC might interact in different contexts and also PKCδ, ε, and ζ might have different activation mechanisms in hPS cells. It is needed further investigation in future.

GSK-3β is inhibited by phosphorylation stimulated by the canonical Wnt signal pathway, which is followed by the accumulation of β-catenin to the nucleus [49]. From the above findings, it follows that FGF-2 may activate Wnt signaling through PKC leading to differentiation of hPS cells. This conclusion contradicts the findings of previous studies demonstrating that canonical Wnt signaling supports self-renewal of stem cells [50]–[52]. However, it is consistent with a study showing that canonical Wnt signaling does not appear to promote stem cell maintenance, which prevents differentiation of stem cells [53]. On the other hand, some studies have shown a dual function for Wnt signaling in hES cells in that the pathways of self-renewal or differentiation are dependent on the presence of hES cell supporting factors [51]–[52]. Recently, Ding et al. [32] showed that FGF-2 modulates Wnt signaling through AKT/GSK-3β signaling and suggested that the differences in the results could be due to the culture platform. Our findings suggest that GSK-3β activity is regulated by FGF-2 through both PI3K/AKT and PKC pathways. AKT/GSK-3β signaling may support self-renewal whereas PKC/GSK-3β may promote cell differentiation of hPS cells. However, GFX decreased the phosphorylation level of GSK-3β to lower level than non-treatment. GSK-3β signaling might be stimulated also by other signal pathway in hPS cells. Target genes of these pathways and further regulation mechanisms in GSK-3β signaling should be analyzed in future.

TGF-β/activin/nodal pathways are thought to crosstalk with FGF signaling in regulating hPS cells. Vallier et al. [1]–[2], [54] demonstrated that activin/nodal pathway in co-operation with FGF-2 is necessary for the maintenance of pluripotency in hES cells. We recently reported that activin A enhances FGF-2-induced ERK-1/2, which permits neural and mesendodermal differentiation of hES cells [20]. In this study we showed that activin A enhanced FGF-2-induced phosphorylation of not only ERK-1/2 but also GSK-3β. Inhibition of these pathways provided stable culture of hPS cells for long-term. In this study, we used both GFX and U0126 to inhibit these pathways. GFX targeting all of PKCα, β, γ, δ, ε, and ζ had no inhibitory effect on ERK-1/2 pathway although siRNA targeting PKCε or PKCζ decreased it. If more specific inhibitor is developed in future, it would be more useful. To maintain undifferentiated state, balancing among ERK-1/2, PI3K, SMAD, and PKC signal pathways may be required in any culture conditions. KSR of which components are not disclosed in public is known to have BMP-4-like activity [55]. Some components including BMP-4 in KSR together with secreting factors from mouse feeders might regulate PKC/ERK-1/2 signaling. Using our defined conditions, more molecules including growth factors would be screened to detect their accurate effects on hPS cells.

In conclusion, our study suggested that FGF-2 induced PI3K/AKT and MEK/ERK-1/2, but also PKCs in hPS cells. PI3K/AKT promotes cell self-renewal whereas the MEK/ERK-1/2, PKC/ERK-1/2 and PKC/GSK-3β pathways down-regulate hPS cell self-renewal. This study helps to untangle the cross-talk between molecular mechanisms regulating self-renewal and differentiation of hPS cells.

## Materials and Methods

### Chemicals

A chemical library of kinase inhibitors (Biomol, Plymouth Meeting, PA, USA), LY-294002 (Cell Signaling Technology, Beverly, MA, USA), BIO (Merck, Darmstadt, Germany), U0126 (Promega, Madison, WI, USA), Y-27632 (Wako Pure Chemical, Osaka, Japan), PMA (Sigma, St. Louis, MO, USA), 4α-PMA (Promega) and Gö6976 (Sigma) were dissolved in dimethyl sulfoxide (DMSO). LiCl (Sigma) and GF109203X hydrochloride (Sigma) were dissolved in water.

### PKC inhibitory peptides

Membrane-permeable PKCδ inhibitory peptide δV1-1 (SFNSYELGSL: amino acids 8-17 of PKCδ) or PKCε inhibitory peptide εV1-2 (EAVSLKPT: amino acids 14-21 of PKCε) were designed according to the method of Mochly-Rosen [56]–[57]. The peptides were custom-synthesized by Sigma (purified to >95% by HPLC). Myristoylated PKCα, β, and γ inhibitory peptide and myristoylated PKCζ inhibitory peptide were purchased from Promega and Calbiochem (Darmstadt, Germany), respectively.

### Cell culture

The hES cell lines, H9 [10], [31] (WA09, WISC Bank, WiCell Research Institute, Madison, WI, USA) and KhES-4 (provided by Kyoto University, Kyoto, Japan), and hiPS cell lines, 201B7 [26] (provided by Dr. Shinya Yamanaka, Kyoto University) and Tic (JCRB1331, JCRB Cell Bank, Osaka, Japan) [33], [58] were routinely maintained on mitomycin C-inactivated mouse embryo fibroblast feeder cells (MEF, Millipore Co., Billerica, MA, USA) in an KSR-based medium supplemented with 5 ng/ml (H9, khES-4), 4 ng/ml (201B7) or 10 ng/ml (Tic) human recombinant FGF-2 (Katayama Kagaku Kogyo LTD., Osaka, Japan) previously described [10]. Human ES cells were used following the Guidelines for utilization of human embryonic stem cells of the Ministry of Education, Culture, Sports, Science and Technology of Japan after approval by the institutional ethical review board at National Institute of Biomedical Innovation. The cells were passaged with 1 mg/ml dispase (Roche, Mannheim, Germany) in DMEM/F12 medium and a plastic scraper (Sumitomo Bakelite Co., LTD Tokyo, Japan). The cells were split at a ratio of 1∶5–1∶8 every 5 days.

### Human ES/iPS cell culture in feeder-free and growth factor defined serum-free medium

Prior to culture in feeder-free conditions, the medium was changed from the KSR-based medium to a growth factor-defined serum-free hESF9 medium [8] (Table S1). Two days after the medium change, the cells were harvested with 1 mg/ml dispase or TrypLE (Invitrogen), and reseeded on plastic plates coated with bovine FN (Sigma, 2 µg/cm^2^) [21]. For long-term culture, hPS cells were maintained on FN in hESF9 medium supplemented with 10 ng/ml human recombinant activin A (R&D Systems Minneapolis, MN, USA) in the presence of both 5 µM U0126 [20], and 5 µM GFX, designated hESF9a_2i_ medium. The medium was changed every day.

### Single hPS cell culturing with two inhibitors

hPS cells were dissociated with TrypLE (Invitrogen) into single cells, and seeded on a 6-well plate coated with FN at the cell density of 1×10^6^ cells/well in hESF9, or supplemented with 10 ng/ml activin A, 5 µM U0126, or 5 µM GFX. The medium was changed every day.

### Quantitative ALP activity-based high-throughput screening assay

The hPS cells were dissociated with accutase into single cells and seeded at 5×10^4^ cells/well on a 96-well plate coated with FN (FN, 2 µg/cm^2^) in hESF9 medium. Each compound in the chemical library was added at 2.5 µM to each well. After further 5 days-culture, the cells were washed with 3-[4-(2-Hydroxyethyl)-1-piperazinyl] propanesulfonic acid (EPPA) buffer (30 mM, pH 8.2). Fluorescence ALP substrate (0.2 mM, 4-methylumbelliferyl phosphate) [59] in EPPS buffer was added into the wells. After incubation for 30 min at 37°C, EPPS buffer (100 mM, pH 7.7) supplemented with 1 M K_2_HPO_4_ was add to terminate the enzyme reaction. The amount of 4-methylumbelliferone (4-MeU) produced via the enzyme reaction was measured with a fluorescence microplate reader (Gemini EM, Molecular Devices, Menlo Park, CA). The specific activity of ALP was quantified by reference to a standard fluorescence curve generated with known concentrations of 4-MeU (Sigma).

### Colony formation assay

Dissociated single hPS cells were seeded at 10,000–250,000 cells/well on a 6-well plate coated with FN (2 µg/cm^2^) in hESF9 medium supplemented with and without 1 µM GFX. After 5-days-culture, the colonies were fixed in 4.5 mM citric acid, 2.25 mM sodium citrate, 3.0 mM sodium chloride, 65% methanol, and 3% formaldehyde for 5 min, and stained with ALP fast-red substrate (Sigma) for 15 min at room temperature.

### Immunocytochemistry

Immunocytochemistry was performed as described previously [20], [60]. The image analysis was performed with In Cell analyzer 2000 and Developer tool box software (GE Healthcare, Little Chalfont, Buckinghamshire, UK), or a confocal microscope (Carl Zeiss). The primary and secondary antibodies used were listed in Table S2.

### Western blotting

Western blots were performed as described previously [8], [20], [60]. Protein (2 µg/lane) was separated by 12.5% SDS-PAGE and transferred to polyvinylidene fluoride (PVDF) membranes (Millipore). The membranes were reacted with primary antibodies, peroxidase-conjugated secondary antibodies, and ECL Plus reagent (GE Healthcare). Protein bands were visualized using LAS-4000 imager (Fujifilm, Tokyo, Japan). The primary antibodies used were listed in Table S2.

### AlphaScreen assay

AlphaScreen® SureFire® Cell-based Assay (Perkin-Elmer, Waltham, MA, USA) was performed to measure phosphorylation of AKT-1/2/3, ERK-1/2, and GSK-3β in the cells according to the manufacturer's instructions. Materials used were listed in Table S2. The fluorescence signal was measured using an EnSpire™ plate reader (PerkinElmer).

### Gene expression analysis

Total RNA extracted from cultured cells using RNeasy Mini kit (Qiagen, Valencia, CA, USA) were treated with DNase I to remove any genomic contamination, and reverse-transcribed using Superscript VILO cDNA synthesis kit (Invitrogen) according to the manufacturer's instructions. For RT-PCR, PCR products were amplified with AmpliTaq Gold DNA polymerase (Applied Biosystems, Foster City, CA, USA), following manufacturer's instruction. The DNA was separated by gel electrophoresis and visualized under ultraviolet light for photography. For quantitative real-time RT-PCR, PCR was performed based on the TaqMan or the SYBR Green gene expression technology in a 7300 Real Time PCR System (Applied Biosystems), following manufacturer's instruction. Threshold cycles were normalized to the housekeeping gene GAPDH and translated to relative values. Specific primers used are listed in Tables S3 and S4. For PCR-array, TaqMan low-density human stem cell pluripotency card PCR array (Applied Biosystems, Foster City, CA) was performed as previously described [61]. Expression levels were all normalized against the housekeeping gene β-actin. The relative expression levels of each gene in embryoid bodies were compared to the levels in H9 hES cells or 201B7 hiPS cells grown on feeders in KSR-based medium.

### Transfections with siRNA

Transfections with siRNA were performed using Dharmafect1 (Dharmacon, Chicago, USA) as previously described [62]. Prior to transfection, the hiPS cells were incubated with ROCK inhibitor Y-27632 (10 µM) for 1 hour and dissociated with TrypLE (Invitrogen) and pelleted by centrifugation. To prepare siRNA/lipid solutions, 50 pmol of siRNAs were diluted in 100 µl of hESF9 medium. In a separate tube, 6 µl of Dharmafect1 was diluted in 100 µl of hESF9 medium. The solution of the two tubes were mixed and incubated at room temperature for 20 mins. The resulting 200 µl of siRNA/lipid solution in hESF9 medium was used to resuspend the cell pelleted containing from 1×10^4^ to 1×10^5^ cells, and suspension incubated at room temperature for 10 min. After incubation, 1.5 ml of prewarmed hESF9 medium containing ROCK inhibitor (10 µM) was added and the suspension transferred into a FN-coated well of 24-well or 6-well plate, followed by culture for 24 hour. After recovery in fresh hESF9 medium, cells were transfected again at 24 hours. Total RNAs or proteins were extracted for analysis 72 hours after the fast transfection. siRNAs were listed as Table S4.

### Live cell imaging analysis

After seeded on a 6-well plate coated with FN, the cells were incubated in a live cell imaging system, BioStation CT (Nikon Instruments Inc., Tokyo, Japan) at 37°C 10% CO_2_. The images were captured every 12 hours and analyzed by a soft ware CL-Quant (Nikon Instruments Inc.).

### Cell Growth

The cells were inoculated on a 6-well plate coated with FN at the cell density of 250,000 cells/well in hESF9 medium including 10 ng/ml FGF-2, supplemented with 0.1% DMSO, GFX in H_2_O, or Gö6976 in DMSO. After 5 days culture, the cell numbers were counted by Coulter Counter (Beckman Coulter, Inc).

### Flow cytometry

Flow cytometry was performed as described previously [61] with a FACS Canto flow cytometer (BD Biosciences). The primary antibodies used were listed in Table S2.

### In vitro cell differentiation

In vitro differentiation was induced by the formation of embryoid bodies as described previously [61]. Floating embryoid bodies were maintained in DMEM with 10% FCS for more 14 days.

### Teratoma formation

The cells were harvested by dispase treatment, collected into tubes, and centrifuged, and the pellets were suspended in DMEM supplemented ROCK inhibitor. The cells from a confluent one-well in 6-well plate were injected to the rear leg muscle or thigh muscle of a SCID (C.B-17/lcr-scid/scidJcl) mouse (CLEA japan, Tokyo, Japan). Nine weeks after injection, tumors were dissected, weighted, and fixed with 10% formaldehyde Neutral Buffer Solution (Nacalai tesque, Kyoto, Japan). Paraffin-embedded tissue was sliced and stained with hematoxylin and eosin. All animal experiments were conducted in accordance with the guidelines for animal experiments of the National Institute of Biomedical Innovation, Osaka, Japan.

### Karyotype analysis

Log phase hPS cells (day 3–4 after subculture) were treated with metaphase arresting solution (Genial Genetic Solutions Ltd., Cheshire, UK) for 5 hr. The treated hPS cells were collected with 0.1% EDTA and processed according to the quality control protocol in the JCRB Cell Bank (http://cellbank.nibio.go.jp/cellbank.html). Chromosome numbers were counted in 20 metaphases, and G-banding karyotype analysis was performed on 20 metaphase cells per sample.

## Supporting Information

Figure S1 **The phosphorylation of AKT, GSK-3β, and ERK-1/2 was confirmed by western blot analysis using an antibody to AKT, GSK-3β, and ERK-1/2 and their phosphorylated forms.** Each gel image is a representative of independent three to five experiments. (**A**) Time course of phosphorylation level of AKT, GSK-3β, and ERK-1/2. H9 hES cells were stimulated with FGF-2 (100 ng/ml) with or without GFX (5 µM) for 180 minutes after overnight starvation of FGF-2 and insulin. (**B**) Effect of inhibitors on phosphorylation level of AKT, GSK-3β, and ERK-1/2. After starvation of FGF-2 and insulin overnight, 201B7 hiPS cells were stimulated with FGF-2 (100 ng/ml) for 15 min with LY294002, GFX, U0126, or BIO or without GFX (5 µM). (**C**) Effect of BMP-4 or activin A on phosphorylation level of AKT, GSK-3β, and ERK-1/2. After starvation of FGF-2 and insulin overnight, 201B7 hiPS cells were stimulated with with FGF-2 (100 ng/ml), BMP-4 (10 ng/ml) or activin A (100 ng/ml). (**D**) Effect of addition of activin A with and without inhibitors on phosphorylation level of AKT, GSK-3β, and ERK-1/2. After starvation of FGF-2 and insulin overnight, H9 hES cells were stimulated with FGF-2 (10 ng/ml) and activin A (10 or 100 ng/ml) together with U0126 (5 µM) and GFX (5 µM) or Gö6976 (5 µM) for 15 minutes. (**E**) Effect of GFX concentration on phosphorylation level of AKT, GSK-3β, and ERK-1/2. After starvation of FGF-2 and insulin overnight, H9 hES cells were stimulated with FGF-2 (100 ng/ml) with GFX at 1∼10 µM. The phosphorylation levels in the cells were measured by AlphaScreen® SureFire® assay kit. The values of the y-axis are the ratio of each phosphorylation to each total signal protein. The data are represented as means ± SD (n = 3). *P<0.05.
(TIF)

Figure S2 **Summary of the result of the effect of PI3K, MEK-1/2, or PKCs inhibitor on FGF-2-induced phosphorylation of AKT, GSK-3β, and ERK-1/2.**
(TIF)

Figure S3 **Knockdown efficacy and effect of siRNA targeting PKCδ, ε, and ζ.** (**A**) Total RNAs were extracted for analysis 72 hours after the fast transfected to 201B7 iPS cells. The efficacy of siRNA was evaluated by quantitative RT-PCR. siRNAs and primers were listed as Table S4. (**B**) Summary of the result of the PKCδ-, PKCε-, PKCζ-knockdown effect on phosphorylation of GSK-3β and AKT in FGF-2 signaling.
(TIF)

Figure S4 **Effect of inhibitory peptides for PKCs on phosphorylation level of ERK-1/2.** After starvation of FGF-2 and insulin, the H9 hES cells (right panel) or the 201B7 iPS cells (left panel) were stimulated with FGF-2 (100 ng/ml) for 15 mins with indicated combination of membrane-permeable specific inhibitory peptides for PKC isoforms; PKCα, β, and γ inhibitory peptide (50 µM), PKCδ inhibitory peptide (50 µM), PKCε inhibitory peptide (50 µM), or PKCζ inhibitory peptide (20 µM). The phosphorylation levels in the cells were measured by AlphaScreen® SureFire® assay kit. The values of the y-axis are the ratio of each phosphorylation to each total signal protein. The data are represented as means ± SD (n = 3). *P<0.05.
(TIF)

Figure S5 **Culture of hiPS cells in the hESF9 + activin A + 2i or the hESF9 + activin A + GFX conditions.** (**A**) Phase-contrast image of H9 hES cells serially cultured in hESF9 + activin A + 2i (hESF9a_2i_) or hESF9 + activin A + GFX mediums at three passages, as described in Figure 5A and 5B. Scale bars, 200 µm. (**B**) Immunocytochemical staining for OCT3/4 expression of H9 cells cultured as described (A). The H9 hES cells stained with anti-OCT3/4 antibody were visualized with Alexa Fluor 488 (green). Nuclei were stained with Hoechst 33342 (blue). Scale bars, 50 µm. (**C**) Anti-OCT3/4 staining intensity profiles in the cell population grown in the hESF9 + activin A + 2i or the hESF9 + activin A + GFX conditions were analyzed by IN Cell image analyzer (lower panels). Antigen histogram (red); control histogram (green); Y axis is cell numbers and X axis is fluorescence intensity for anti-OCT3/4 antibody.
(TIF)

Figure S6 **Immunocytochemical staining of H9, KhES-4, 201B7, and Tic hPS cells for TRA-1-60.** The cells grown on FN in hESF9a_2i_ as described in Figure 5C were stained with TRA-1-60 antibody and Alexa Fluor 647-conjugated secondary antibody. Nuclei were stained with Hoechst 33342 (blue). Scale bars, 200 µm.
(TIF)

Figure S7 **Long-term culture of hiPS cells in the hESF9a_2i_ medium.** Human iPS 201B7 cells were cultured on FN in hESF9a_2i_ medium serially for more than 30 passages. The cells were split at a ratio of 1∶3–1∶5 every five days. (**A**) Phase-contrast image of 201B7 hiPS cells cultured on FN in hESF9a_2i_ medium. (**B**) A comparison of the growth of 201B7 cells in hESF9a_2i_ medium or KSR-based media. The cells were seeded on feeders in KSR-based medium (closed circles) or on FN in hESF9a_2i_ medium (open circles; mean + s.d. of three experiments. Cell numbers were counted every 2 days. (**C**) Immunocytochemical staining for SSEA-1, SSEA-4, TRA-1-60 and TRA-1-81 (red) expression of 201B7 cells (passage 10) cultured on FN in hESF9a_2i_. Nuclei were stained with Hoechst 33342 (blue). Scale bars, 200 µm. (**D**) FACS profiles for SSEA-1, SSEA-4, TRA-1-60, TRA-1-81, TRA-2-54, A2B5, CD90, and HLA-Class1 expression of hiPS 201B7 cells (passage 22) cultured on FN in hESF9a_2i_ medium. Antigen histogram (red); control histogram (green); the horizontal bar indicates the gating used to score the percentage of antigen-positive cells.
(TIF)

Figure S8 **Long-term culture of hES cells in the hESF9a_2i_ medium.** Human ES H9 cells were cultured on FN in hESF9a_2i_ medium serially for more than 30 passages. The cells were split at a ratio of 1∶3–1∶5 every five days. (**A**) Phase-contrast image of H9 hES cells cultured on FN in hESF9a_2i_ medium. (**B**) A comparison of the growth of H9 hES cells (passage 13, 16, and 17) in hESF9a_2i_ (open circles) or KSR-based media (closed circles). Mean + s.d. of three experiments. (**C**) Immunocytochemical staining for SSEA-1, SSEA-4, TRA-1-60, TRA-1-81, TRA-2-54, A2B5, CD90, and HLA-Class1 expression (red) in H9 hES cells (passage 13). Nuclei were stained with Hoechst 33342 (blue). (**D**) FACS profiles of H9 hES cells (passage 14). Antigen histogram (red); control histogram (green). Scale bars = 200 µm.
(TIF)

Figure S9 **Karyotype analysis and differentiation potential of H9 hES cells and 201B7 hiPS cells maintained in hESF9a_2i_ conditions.** (**A**) Karyotype analysis of H9 hES cells at passage 15 and 201B7 hiPS cells at passage 21, showing a normal diploid 46, xx karyotype. (**B**) Heat-map of gene expression in H9 hES cells (at passage 10–13) and 201B7 hiPS cells (at passage 10–20) those during in vitro differentiation in triplicate experiments (Sample No. 3–5). TaqMan low density PCR arrays (Applied BioSystems) were performed as previously described [61]. Expression levels were all normalized against β-ACTIN. The relative level of each gene expression were generated from the undifferentiated H9 hES cell or 201B7 hiPS cells cultured on mitomycin-inactivated mouse embryonic fibroblasts (MEF) in KSR-based medium (Sample No. 1–2). Heat-map colors (red for up-regulation, blue for down-regulation) depict gene expression. (**C**) Teratomas derived from H9 hES cells at passage 44 or 201B7 iPS cells at passage 26 maintained in hESF9a_2i_ conditions.
(TIF)

Table S1 **The composition of media used for serum-free culture.** * The composition of the basal medium, ESF for culturing mouse ES cells, is described in Furue et al., 2005 [22]. ** hESF9 medium is described in Furue et al., 2008 [8]. *** hESF9a medium is described in Hayashi and Furue et al., 2010 [23].
(DOC)

Table S2 **A list of the used antibodies.**
(DOC)

Table S3 **A list of the used primers for RT-PCR.**
(DOC)

Table S4 **A list of the used primers for qRT-PCR and siRNAs.**
(DOC)
