# Supplementary figures and images for: Protein Kinase C Regulates Human Pluripotent Stem Cell Self-Renewal
Source: PLoS One. 2013 Jan 21;8(1):e54122. doi: 10.1371/journal.pone.0054122 (PMC3549959; doi:10.1371/journal.pone.0054122)

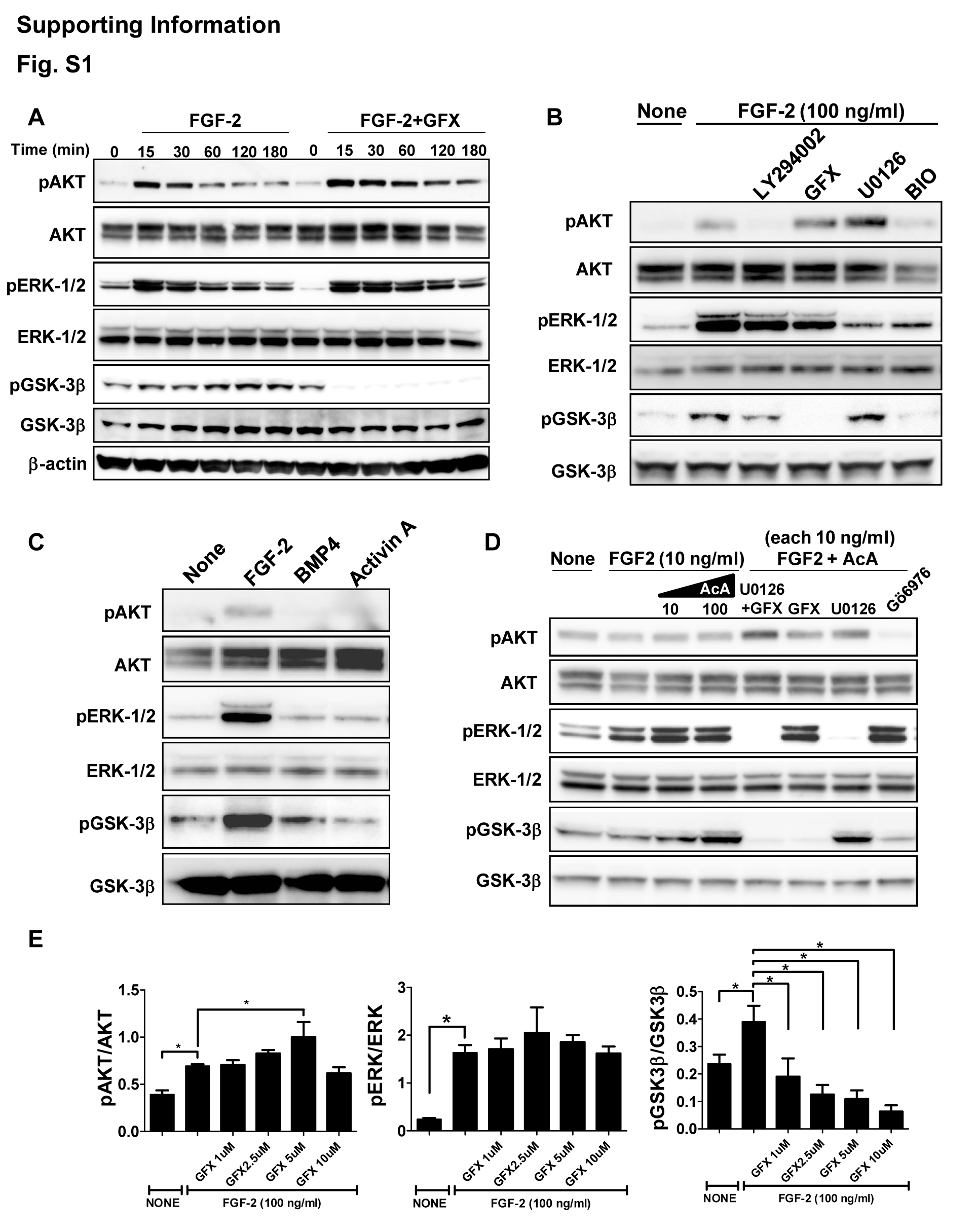

Supplement: Figure S1 — The phosphorylation of AKT, GSK-3β, and ERK-1/2 was confirmed by western blot analysis using an antibody to AKT, GSK-3β, and ERK-1/2 and their phosphorylated forms. Each gel image is a representative of independent three to five experiments. (A) Time course of phosphorylation level of AKT, GSK-3β, and ERK-1/2. H9 hES cells were stimulated with FGF-2 (100 ng/ml) with or without GFX (5 µM) for 180 minutes after overnight starvation of FGF-2 and insulin. (B) Effect of inhibitors on phosphorylation level of AKT, GSK-3β, and ERK-1/2. After starvation of FGF-2 and insulin overnight, 201B7 hiPS cells were stimulated with FGF-2 (100 ng/ml) for 15 min with LY294002, GFX, U0126, or BIO or without GFX (5 µM). (C) Effect of BMP-4 or activin A on phosphorylation level of AKT, GSK-3β, and ERK-1/2. After starvation of FGF-2 and insulin overnight, 201B7 hiPS cells were stimulated with with FGF-2 (100 ng/ml), BMP-4 (10 ng/ml) or activin A (100 ng/ml). (D) Effect of addition of activin A with and without inhibitors on phosphorylation level of AKT, GSK-3β, and ERK-1/2. After starvation of FGF-2 and insulin overnight, H9 hES cells were stimulated with FGF-2 (10 ng/ml) and activin A (10 or 100 ng/ml) together with U0126 (5 µM) and GFX (5 µM) or Gö6976 (5 µM) for 15 minutes. (E) Effect of GFX concentration on phosphorylation level of AKT, GSK-3β, and ERK-1/2. After starvation of FGF-2 and insulin overnight, H9 hES cells were stimulated with FGF-2 (100 ng/ml) with GFX at 1∼10 µM. The phosphorylation levels in the cells were measured by AlphaScreen® SureFire® assay kit. The values of the y-axis are the ratio of each phosphorylation to each total signal protein. The data are represented as means ± SD (n = 3). *P<0.05. (TIF) [file pone.0054122.s001.tif]

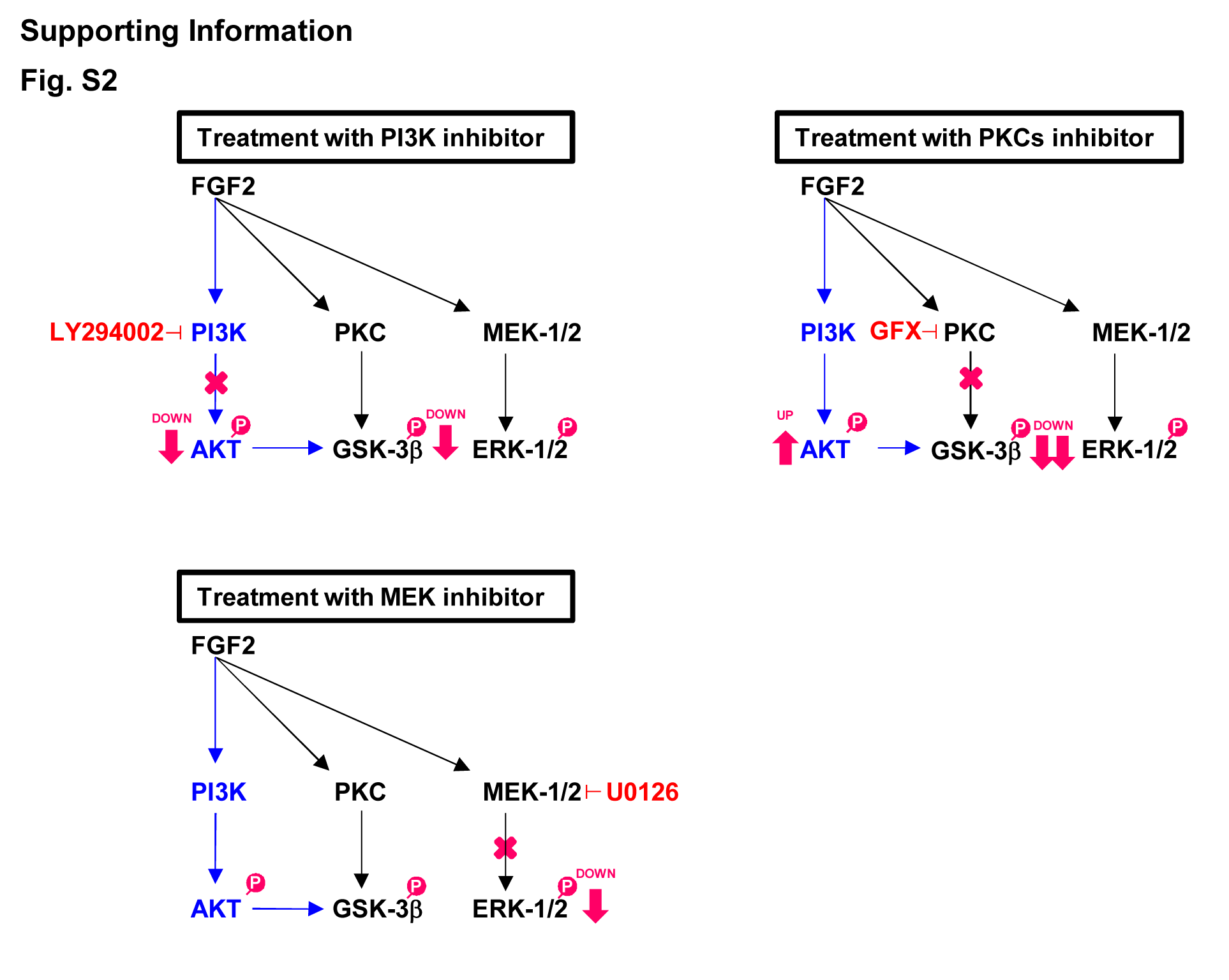

Supplement: Figure S2 — Summary of the result of the effect of PI3K, MEK-1/2, or PKCs inhibitor on FGF-2-induced phosphorylation of AKT, GSK-3β, and ERK-1/2. (TIF) [file pone.0054122.s002.tif]

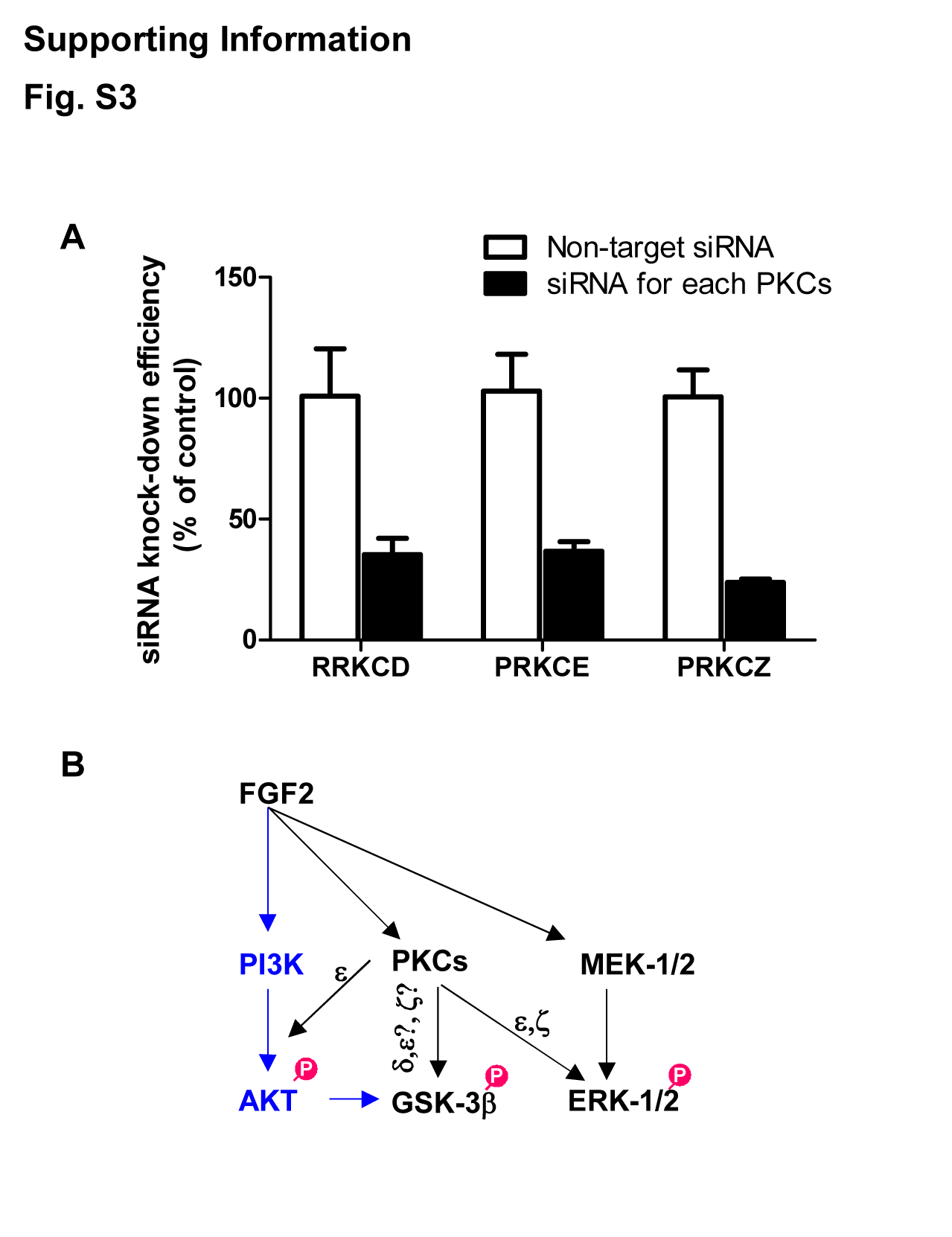

Supplement: Figure S3 — Knockdown efficacy and effect of siRNA targeting PKCδ, ε, and ζ. (A) Total RNAs were extracted for analysis 72 hours after the fast transfected to 201B7 iPS cells. The efficacy of siRNA was evaluated by quantitative RT-PCR. siRNAs and primers were listed as Table S4. (B) Summary of the result of the PKCδ-, PKCε-, PKCζ-knockdown effect on phosphorylation of GSK-3β and AKT in FGF-2 signaling. (TIF) [file pone.0054122.s003.tif]

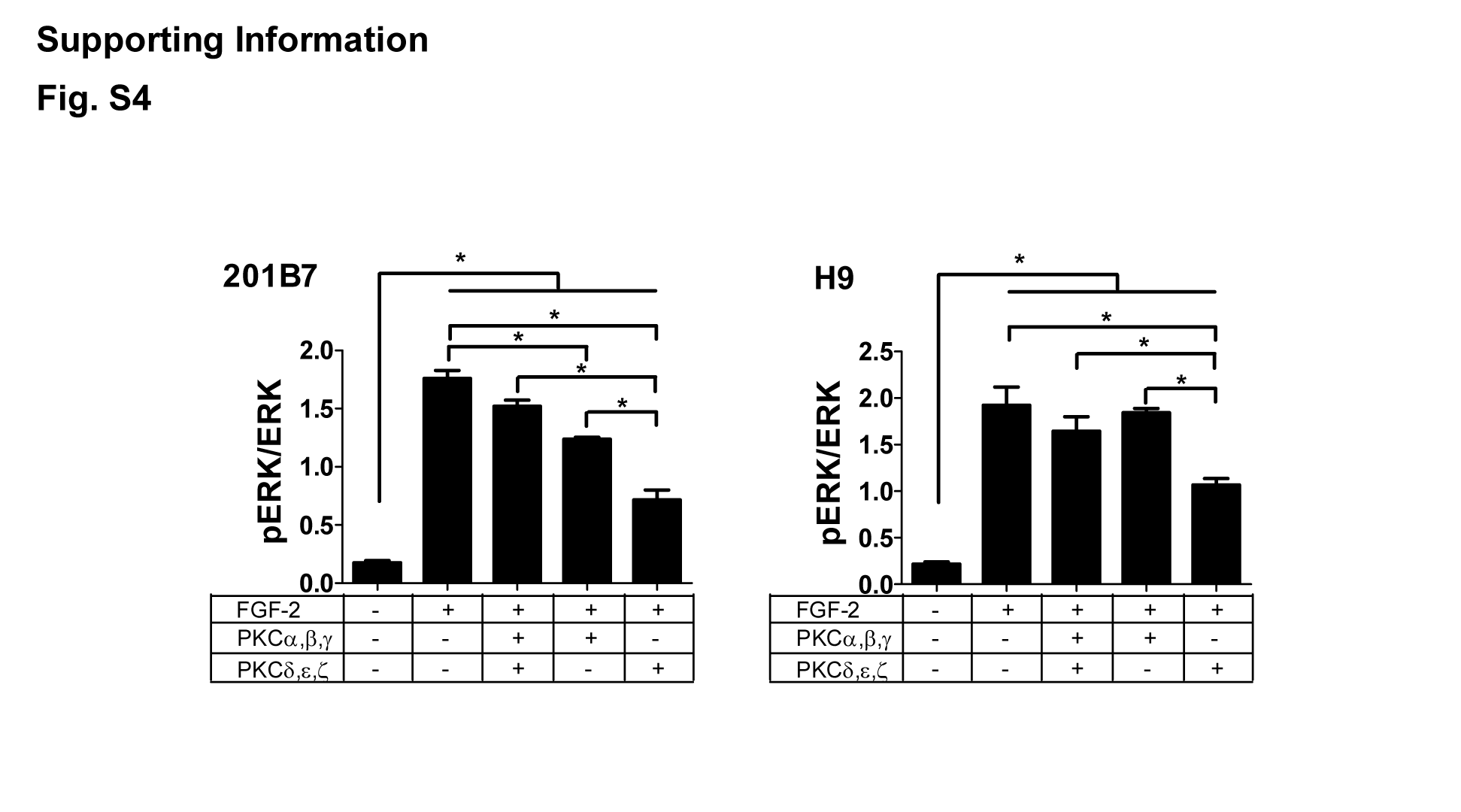

Supplement: Figure S4 — Effect of inhibitory peptides for PKCs on phosphorylation level of ERK-1/2. After starvation of FGF-2 and insulin, the H9 hES cells (right panel) or the 201B7 iPS cells (left panel) were stimulated with FGF-2 (100 ng/ml) for 15 mins with indicated combination of membrane-permeable specific inhibitory peptides for PKC isoforms; PKCα, β, and γ inhibitory peptide (50 µM), PKCδ inhibitory peptide (50 µM), PKCε inhibitory peptide (50 µM), or PKCζ inhibitory peptide (20 µM). The phosphorylation levels in the cells were measured by AlphaScreen® SureFire® assay kit. The values of the y-axis are the ratio of each phosphorylation to each total signal protein. The data are represented as means ± SD (n = 3). *P<0.05. (TIF) [file pone.0054122.s004.tif]

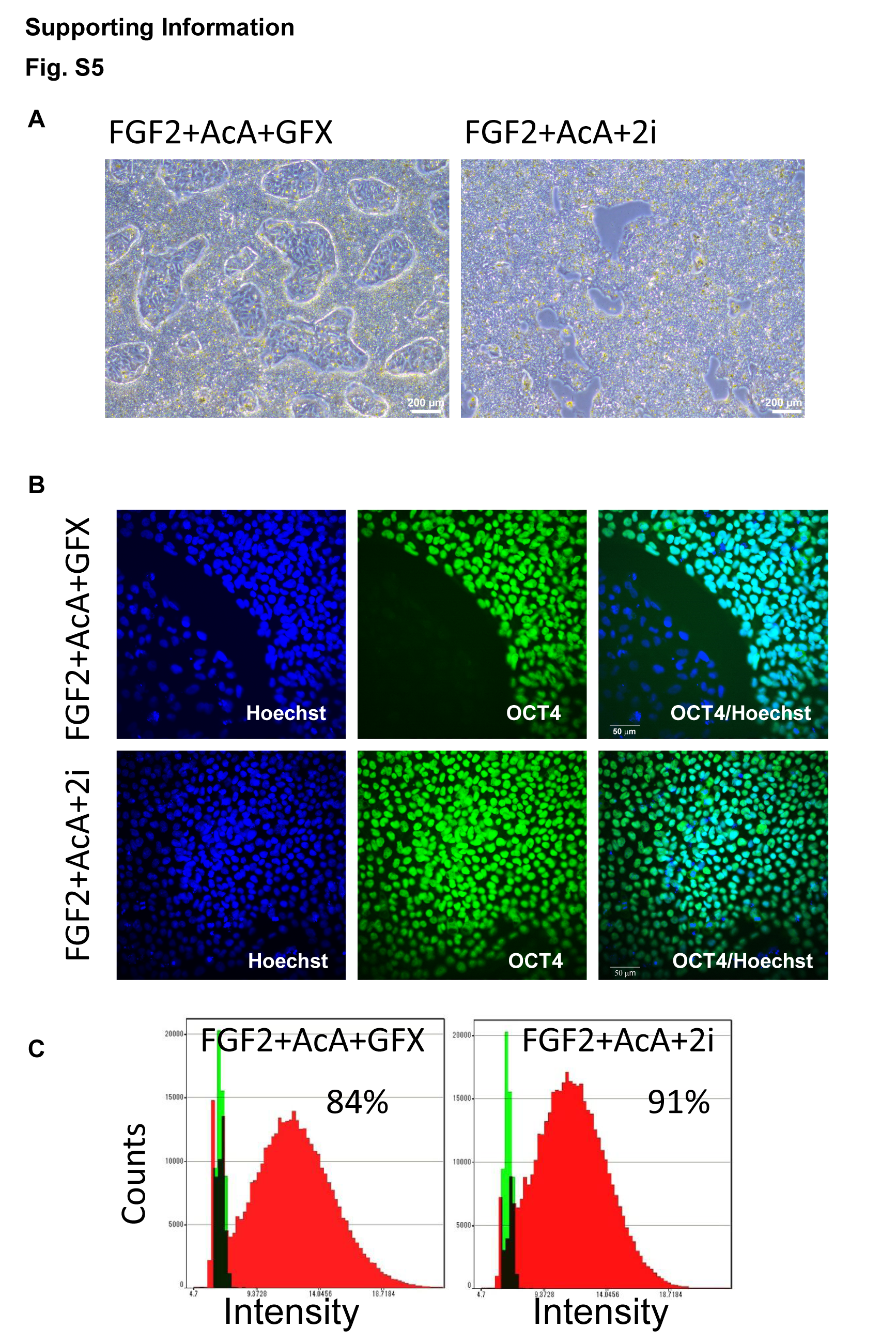

Supplement: Figure S5 — Culture of hiPS cells in the hESF9 + activin A + 2i or the hESF9 + activin A + GFX conditions. (A) Phase-contrast image of H9 hES cells serially cultured in hESF9 + activin A + 2i (hESF9a2i) or hESF9 + activin A + GFX mediums at three passages, as described in Figure 5A and 5B. Scale bars, 200 µm. (B) Immunocytochemical staining for OCT3/4 expression of H9 cells cultured as described (A). The H9 hES cells stained with anti-OCT3/4 antibody were visualized with Alexa Fluor 488 (green). Nuclei were stained with Hoechst 33342 (blue). Scale bars, 50 µm. (C) Anti-OCT3/4 staining intensity profiles in the cell population grown in the hESF9 + activin A + 2i or the hESF9 + activin A + GFX conditions were analyzed by IN Cell image analyzer (lower panels). Antigen histogram (red); control histogram (green); Y axis is cell numbers and X axis is fluorescence intensity for anti-OCT3/4 antibody. (TIF) [file pone.0054122.s005.tif]

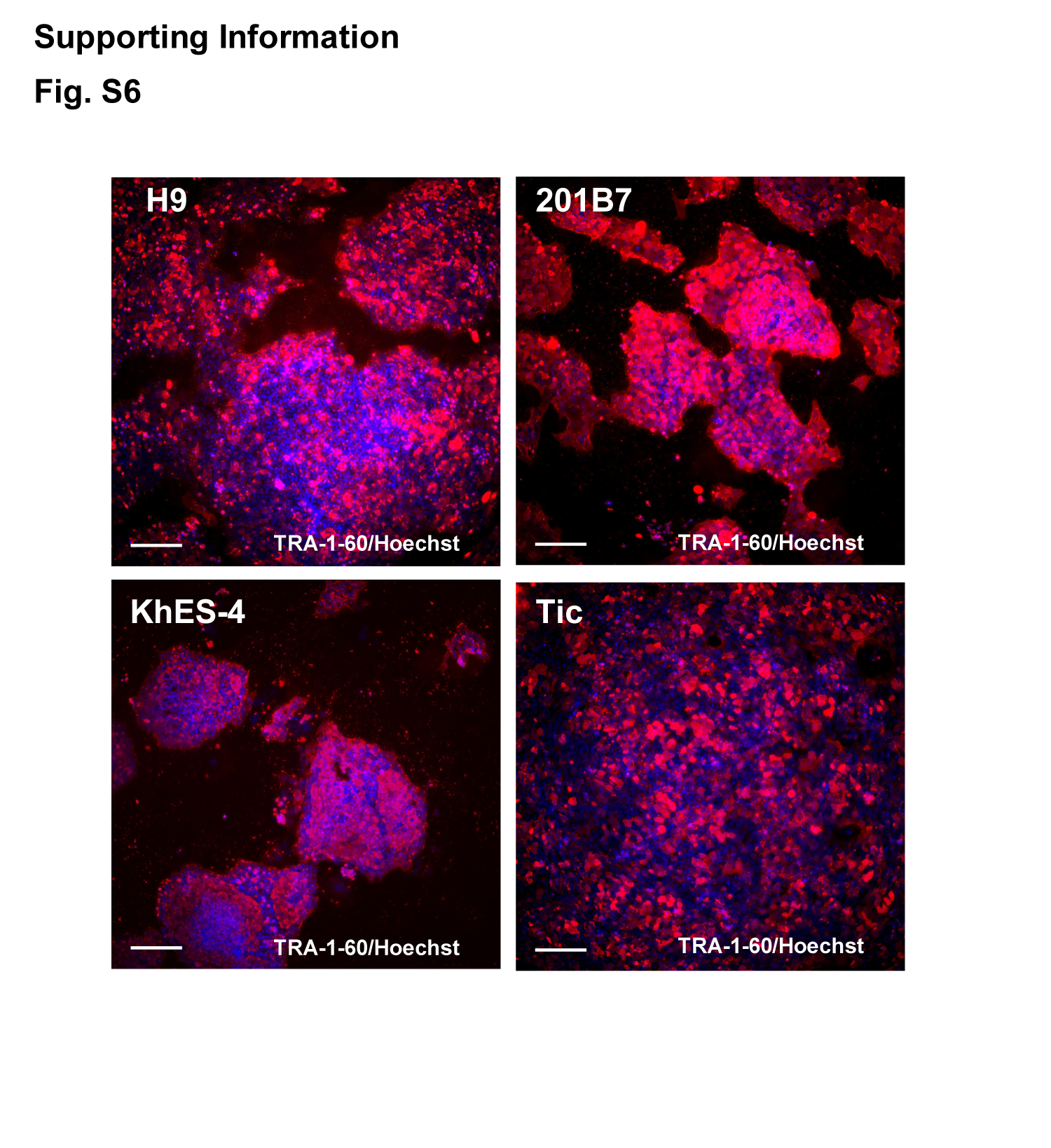

Supplement: Figure S6 — Immunocytochemical staining of H9, KhES-4, 201B7, and Tic hPS cells for TRA-1-60. The cells grown on FN in hESF9a2i as described in Figure 5C were stained with TRA-1-60 antibody and Alexa Fluor 647-conjugated secondary antibody. Nuclei were stained with Hoechst 33342 (blue). Scale bars, 200 µm. (TIF) [file pone.0054122.s006.tif]

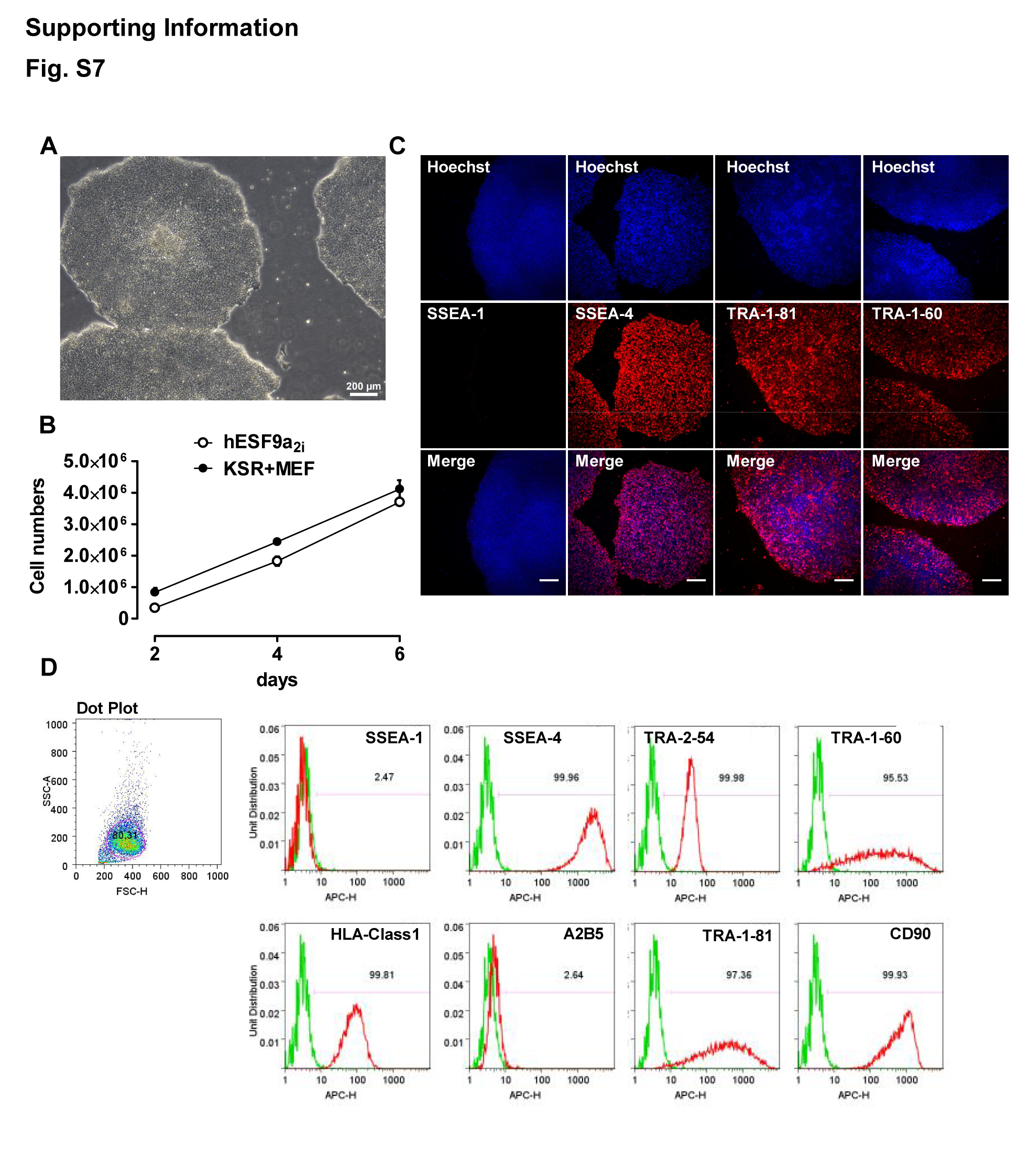

Supplement: Figure S7 — Long-term culture of hiPS cells in the hESF9a2i medium. Human iPS 201B7 cells were cultured on FN in hESF9a2i medium serially for more than 30 passages. The cells were split at a ratio of 1∶3–1∶5 every five days. (A) Phase-contrast image of 201B7 hiPS cells cultured on FN in hESF9a2i medium. (B) A comparison of the growth of 201B7 cells in hESF9a2i medium or KSR-based media. The cells were seeded on feeders in KSR-based medium (closed circles) or on FN in hESF9a2i medium (open circles; mean + s.d. of three experiments. Cell numbers were counted every 2 days. (C) Immunocytochemical staining for SSEA-1, SSEA-4, TRA-1-60 and TRA-1-81 (red) expression of 201B7 cells (passage 10) cultured on FN in hESF9a2i. Nuclei were stained with Hoechst 33342 (blue). Scale bars, 200 µm. (D) FACS profiles for SSEA-1, SSEA-4, TRA-1-60, TRA-1-81, TRA-2-54, A2B5, CD90, and HLA-Class1 expression of hiPS 201B7 cells (passage 22) cultured on FN in hESF9a2i medium. Antigen histogram (red); control histogram (green); the horizontal bar indicates the gating used to score the percentage of antigen-positive cells. (TIF) [file pone.0054122.s007.tif]

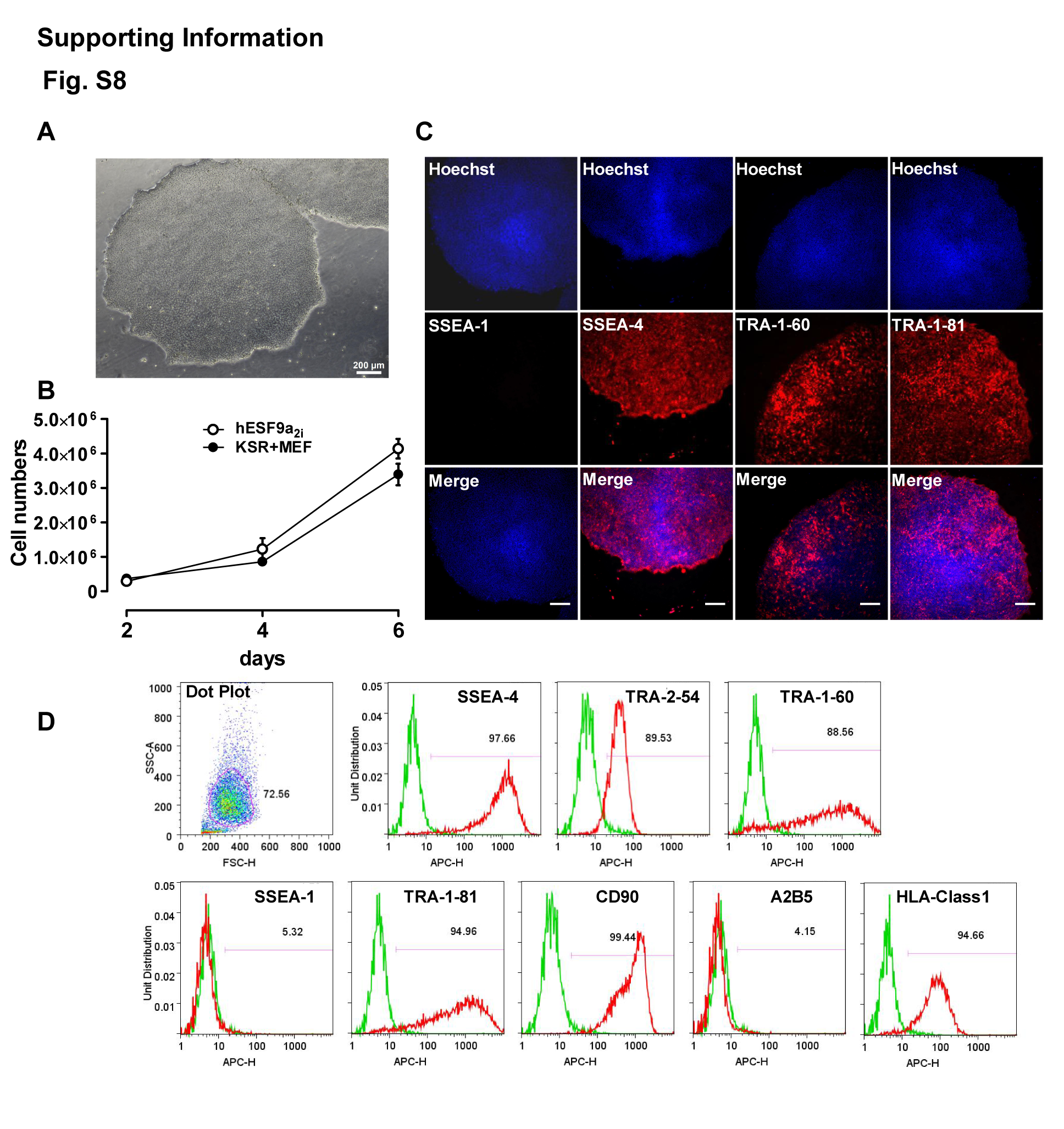

Supplement: Figure S8 — Long-term culture of hES cells in the hESF9a2i medium. Human ES H9 cells were cultured on FN in hESF9a2i medium serially for more than 30 passages. The cells were split at a ratio of 1∶3–1∶5 every five days. (A) Phase-contrast image of H9 hES cells cultured on FN in hESF9a2i medium. (B) A comparison of the growth of H9 hES cells (passage 13, 16, and 17) in hESF9a2i (open circles) or KSR-based media (closed circles). Mean + s.d. of three experiments. (C) Immunocytochemical staining for SSEA-1, SSEA-4, TRA-1-60, TRA-1-81, TRA-2-54, A2B5, CD90, and HLA-Class1 expression (red) in H9 hES cells (passage 13). Nuclei were stained with Hoechst 33342 (blue). (D) FACS profiles of H9 hES cells (passage 14). Antigen histogram (red); control histogram (green). Scale bars = 200 µm. (TIF) [file pone.0054122.s008.tif]

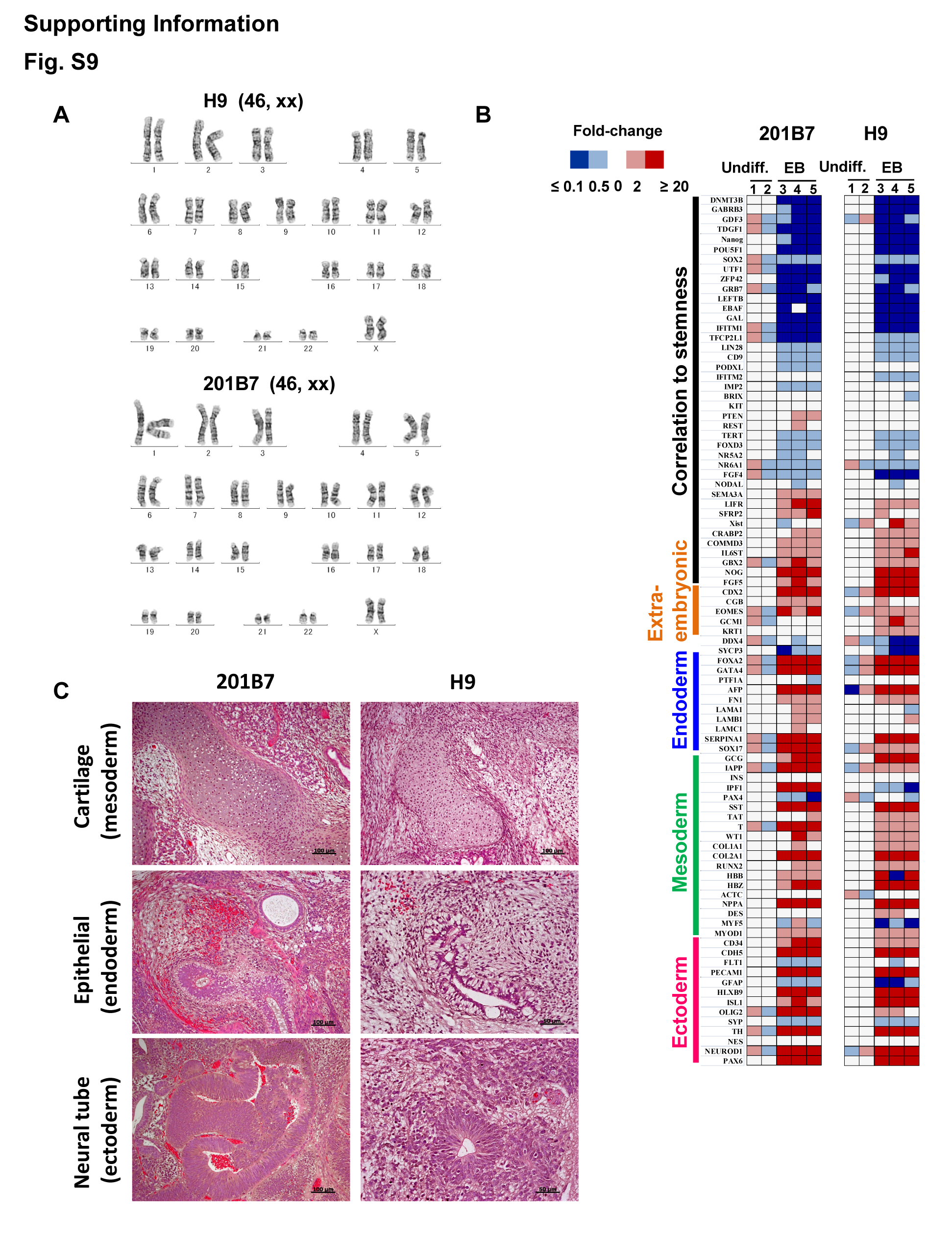

Supplement: Figure S9 — Karyotype analysis and differentiation potential of H9 hES cells and 201B7 hiPS cells maintained in hESF9a2i conditions. (A) Karyotype analysis of H9 hES cells at passage 15 and 201B7 hiPS cells at passage 21, showing a normal diploid 46, xx karyotype. (B) Heat-map of gene expression in H9 hES cells (at passage 10–13) and 201B7 hiPS cells (at passage 10–20) those during in vitro differentiation in triplicate experiments (Sample No. 3–5). TaqMan low density PCR arrays (Applied BioSystems) were performed as previously described [61]. Expression levels were all normalized against β-ACTIN. The relative level of each gene expression were generated from the undifferentiated H9 hES cell or 201B7 hiPS cells cultured on mitomycin-inactivated mouse embryonic fibroblasts (MEF) in KSR-based medium (Sample No. 1–2). Heat-map colors (red for up-regulation, blue for down-regulation) depict gene expression. (C) Teratomas derived from H9 hES cells at passage 44 or 201B7 iPS cells at passage 26 maintained in hESF9a2i conditions. (TIF) [file pone.0054122.s009.tif]
