# Supplementary material for: Protein Kinase C Regulates Human Pluripotent Stem Cell Self-Renewal
Source: PLoS One. 2013 Jan 21;8(1):e54122. doi: 10.1371/journal.pone.0054122 (PMC3549959; doi:10.1371/journal.pone.0054122)
Supplement: Table S1 — The composition of media used for serum-free culture. * The composition of the basal medium, ESF for culturing mouse ES cells, is described in Furue et al., 2005 [22]. ** hESF9 medium is described in Furue et al., 2008 [8]. *** hESF9a medium is described in Hayashi and Furue et al., 2010 [23]. (DOC) [file pone.0054122.s010.doc]

### Supporting Information Table

### Table S1. The composition of media used for serum-free culture.

|  | | hESF9** | hESF9a*** | hESF9a_2i_ |
| --- | --- | --- | --- | --- |
| Basal medium | | ESF* | ESF* | ESF* |
| 4-(2-hydroxyethyl)-1-piperazineethanesulfonic acid (HEPES) | | - | - | - |
| L-ascorbic acid-2-phophate (100 μg/ml) | | + | + | + |
| 6 factors | |  |  |  |
|  | Human recombinant insulin (10 μg/ml) | + | + | + |
|  | Human transferrin (5 μg/ml) | + | + | + |
|  | 2-Mercaptoethanol (10 μM) | + | + | + |
|  | 2-Ethanolamine (10 μM) | + | + | + |
|  | Sodium selenite (20 nM) | + | + | + |
|  | Oleic acid conjugated with fatty acid-free bovine serum albumin (9.4 μg/ml) | + | + | + |
| Bovine heparan sulfate sodium salt | | 100 ng/ml | 100 ng/ml | 100 ng/ml |
| Human recombinant fibroblast growth factor-2 (FGF-2) | | 10 ng/ml | 10 ng/ml | 10 ng/ml |
| Human recombinant activin A | | - | 10 ng/ml | 10 ng/ml |
| U0126 | | - | - | 5 μM |
| GF109203X hydrochloride (GFX) | | - | - | 5 μM |
