# Supplementary material for: Protein Kinase C Regulates Human Pluripotent Stem Cell Self-Renewal
Source: PLoS One. 2013 Jan 21;8(1):e54122. doi: 10.1371/journal.pone.0054122 (PMC3549959; doi:10.1371/journal.pone.0054122)
Supplement: Table S2 — A list of the used antibodies. (DOC) [file pone.0054122.s011.doc]

### Table S3. A list of the used primers for RT-PCR.

| Genes | Forward | Reverse |
| --- | --- | --- |
| *PKCα (PRKCA)* | CGAAGATCTGGACCATGGCTGACG | AACGTCGACGCAGGGCCAAGTTTGGCTTT |
| *PKCβ1 (PRKCB1)* | AAACTGCAGCCCCTTATAAGCCAAAAGCT | AACGTCGACCTTGGCTTGAAGTCTTACAC |
| *PKCβ2 (PRKCB2)* | AAACTTGAACGCAAAGAGATCCA | ATCGGTCGAAGTTTTCAGCATT |
| *PKCγ (PRKCG)* | AAGAAAGGCAGGATCCTGGT | AACGTCGACATCCGCACCCGCTCATACAA |
| *PKCδ (PRKCD)* | GGAAGATCTGCAACGGGAGCCCCACTGCA | CGGAATTCAACCCTGATATATCCCAACA |
| *PKCε (PRKCE)* | TGTGGGACTCCTGACTACATAGC | AATAGGTCGTCCTCATTGTCG |
| *PKCη (PRKCH)* | GCAAGATCTGCATGTCGTCTGGCACCCATG | GCAGTCGACCCAATCCCATTTCCTTC |
| *PKCθ (PRKCQ)* | GCAGGATCCCCATGTCGCCATTTCTTCG | GCTGTCGACCAGGAAATGCCCTGAGGCTC |
| *PKCλ (PRKCL)* | GGCTGCATTCTTGCTTTCAGA | CCAAAAATATGAAGCCCAGTAATCA |
| *PKCζ (PRKCZ)* | CCTGCTTCCAGACGACAAGT | AGAACCTGGCGTGCTCCT |
| *PKCμ (PRKCM)* | CATTGGCGAGAAGTCTTTCCG | TCAGGCTCACATAGATGATGACCC |
| *β-actin (ACTB)* | CTGGCACCACACCTTCTACAATG | AATGTCACGCACGATTTCCCGC |
