# Supplementary material for: Protein Kinase C Regulates Human Pluripotent Stem Cell Self-Renewal
Source: PLoS One. 2013 Jan 21;8(1):e54122. doi: 10.1371/journal.pone.0054122 (PMC3549959; doi:10.1371/journal.pone.0054122)
Supplement: Table S3 — A list of the used primers for RT-PCR. (DOC) [file pone.0054122.s012.doc]

### Table S2. A list of the used antibodies.

| Primary or secondary antibody | Company |
| --- | --- |
| OCT3/4 | Catalog No. sc-5279, Santa Cruz Biotechnology, Santa Cruz, CA, USA |
| HLA-class1 | Catalog No. M0736, Dako, Carpinteria, CA, USA. |
| CD90 | Catalog No. 555593, Dako, Carpinteria, CA, USA. |
| SSEA-1 | Catalog No. sc-21702, Santa Cruz Biotechnology, Santa Cruz, CA, USA |
| SSEA-4 | A generous gift from Prof. P.W. Andrews, Sheffield, UK |
| TRA-1-60 | Catalog No. sc-21705, Santa Cruz Biotechnology, Santa Cruz, CA, USA |
| TRA-1-81 | Catalog No. sc-21706, Santa Cruz Biotechnology, Santa Cruz, CA, USA |
| TRA-2-54 | A generous gift from Prof. P.W. Andrews, Sheffield, UK |
| A2B5 | A generous gift from Prof. P.W. Andrews, Sheffield, UK |
| AKT | Catalog No. #4691, Cell Signaling Technology, Beverly, MA, USA. |
| Phosphorylated AKT (Ser473) | Catalog No. #4060, Cell Signaling Technology, Beverly, MA, USA. |
| ERK-1/2 | Catalog No. #4696, Cell Signaling Technology, Beverly, MA, USA. |
| Phosphorylated ERK-1/2 (Thr202/Thr204) | Catalog No. #4370, Cell Signaling Technology, Beverly, MA, USA. |
| GSK-3β | Catalog No. #9315, Cell Signaling Technology, Beverly, MA, USA. |
| Phosphorylated GSK-3β (Ser9) | Catalog No. #9323, Cell Signaling Technology, Beverly, MA, USA. |
| PKCδ | Catalog No. 610397, BD Biosciences, San Diego, CA, USA. |
| Phosphorylated PKCδ at ser 645 (Ser 643 in mouse and rat) | Catalog No. ab47773, Abcam, Cambridge, UK. |
| PKCε | Catalog No. sc-1681, Santa Cruz Biotechnology, Santa Cruz, CA, USA. |
| Phosphorylated PKCε (Ser 729) | Catalog No. #06-821, Upstate Biotechnology, Lake Placid, NY, USA. |
| PKCζ | Catalog No. ab51157, Abcam, Cambridge, UK. |
| Phosphorylated PKCζ (Thr 560) | Catalog No. ab62372, Abcam, Cambridge, UK. |
| Phosphorylated PKCζ (Thr 410) | Catalog No. ab76129, Abcam, Cambridge, UK. |
| AlphaScreen surefireAKT1/2/3 Total assay kit | Catalog No. TGRTAPSHV100, PerkinElmer, CA, USA. |
| AlphaScreen surefireAKT1/2/3 (p-Ser473) assay kit | Catalog No. TGRA4SHV100, PerkinElmer, CA, USA. |
| AlphaScreen surefireERK 1/2 Total assay kit | Catalog No. TGRTESHV100, PerkinElmer, CA, USA. |
| AlphaScreen surefireERK 1/2 (p-Thr202/Tyr204) assay kit | Catalog No. TGRESHV100, PerkinElmer, CA, USA. |
| AlphaScreen surefireGSK3β (p-Ser9) assay kit | Catalog No. TGRGBS500, PerkinElmer, CA, USA. |
| Biotinylated GSK-3β antibody (used for AlphaScreen assay) | Catalog No. AM00066BT-N, Acris Antibodies, CA, USA. |
| GSK-3β(used for AlhaScreen assay) | Catalog No. 9832, Cell Signaling Technology, Beverly, MA, USA. |
| Alexa Fluor 647-conjugated anti-rat IgM | Catalog No. A21248, Invitrogen, Carlsbad, CA, USA. |
| Alexa Fluor 647-conjugated anti-mouse IgG | Catalog No. A21236, Invitrogen, Carlsbad, CA, USA. |
| Alexa Fluor 647-conjugated anti-mouse IgM | Catalog No. A21246, Invitrogen, Carlsbad, CA, USA. |
| Alexa Fluor 488-conjugated anti-mouse IgG | Catalog No. A21200, Invitrogen, Carlsbad, CA, USA. |
| Alexa Fluor 555-conjugated anti-mouse IgM | Catalog No. A21426, Invitrogen, Carlsbad, CA, USA. |
| HRP-conjugated anti-rabbit IgG | Catalog No. 7074, Cell Signaling Technology, Beverly, MA, USA. |
| HRP-conjugated anti-mouse IgG | Catalog No. 715-035-151, Jackson ImmunoResearch Laboratories, West Grove, PA, USA. |
