# Supplementary material for: Protein Kinase C Regulates Human Pluripotent Stem Cell Self-Renewal
Source: PLoS One. 2013 Jan 21;8(1):e54122. doi: 10.1371/journal.pone.0054122 (PMC3549959; doi:10.1371/journal.pone.0054122)
Supplement: Table S4 — A list of the used primers for qRT-PCR and siRNAs. (DOC) [file pone.0054122.s013.doc]

### Table S4. A list of the used primers for qRT-PCR and siRNAs.

| Genes | Sequences (Company) |
| --- | --- |
| *OCT3/4* | 5’-GACAGGGGGAGGGGAGGAGCTAGG-3’5’-CTTCCCTCCAACCAGTTGCCCCAAA-3’ |
| *NANOG* | 5’-TGAACCTCAGCTACAAACAG-3’5’-TGGTGGTAGGAAGAGTAAAG-3’ |
| *T (Brachyury)* | 5’-TGCTTCCCTGAGACCCAGTT-3’5’-GATCACTTCTTTCCTTTGCATCAAG-3’ |
| *FOXA2* | 5’-GGGAGCGGTGAAGATGGA-3’5’-TCATGTTGCTCACGGAGGAGTA-3’ |
| *GAPDH* | 5’-CAAAGTTGTCATGGATGACC-3’5’-CCATGGAGAAGGCTGGGG-3’ |
| *PKCδ (PRKCD)* | AX-003524 (Thermo scientific) |
| *PKCε (PRKCE)* | AX-004653 (Thermo scientific) |
| *PKCζ (PRKCZ)* | AX-003526 (Thermo scientific) |
| *GAPDH* | AX-004253 (Thermo scientific) |
| Non-targeting siRNA (Control) | Mission siRNA universal negative control (SIC-001, Sigma) |
| siRNA targeting *PKCδ (PRKCD)* | L-003524, ON-TARGETplus SMARTpool (Thermo scientific) |
| siRNA targeting *PKCε (PRKCE)* | L-004653, ON-TARGETplus SMARTpool (Thermo scientific) |
| siRNA targeting *PKCζ (PRKCZ)* | L-003526, ON-TARGETplus SMARTpool (Thermo scientific) |
